# Supplementary material for: A prostate-specific membrane antigen activated molecular rotor for real-time fluorescence imaging
Source: Nat Commun. 2021 Sep 15;12:5460. doi: 10.1038/s41467-021-25746-6 (PMC8443597; doi:10.1038/s41467-021-25746-6)
Supplement: Supplementary file 1 — Supplementary Information [file 41467_2021_25746_MOESM1_ESM.pdf]

Supplementary Information

A prostate-specific membrane antigen activated molecular rotor for real-time fluorescence imaging

Jingming Zhang,[1]† *Anastasia Rakhimbekova,*[2]† *Xiaojiang Duan,*[1]† *Qingqing Yin,*[3] *Catherine A. Foss,*[4] *Yan Fan,*[1] *Yangyang Xu,*[5,6,7,8] *Xuesong Li,*[5,6,7,8] *Xuekang Cai,*[1] *Zsofia Kutil,*[2] *Pengyuan Wang,*[9] *Zhi Yang,*[10] *Ning Zhang,*[11] *Martin G. Pomper,*[4] *Yiguang Wang,*[3]* *Cyril Bařinka,*[2]* *Xing Yang*[1,12]*

[1] Department of Nuclear Medicine, Peking University First Hospital, Beijing 100034, China. [2] Institute of Biotechnology of the Czech Academy of Sciences, BIOCEV, Prumyslova 595, 25250 Vestec, Czech Republic. [3] State Key Laboratory of Natural and Biomimetic Drugs, Peking University, Beijing 100191, China. [4] Russell H. Morgan Department of Radiology and Radiological Science, Johns Hopkins University School of Medicine, Baltimore, 21205 Maryland, United States. [5] Department of Urology, Peking University First Hospital, Beijing 100034, China. [6] The Institute of Urology, Peking University, Beijing 100034, China. [7] National Urological Cancer Center, Beijing 100034, China. [8] Beijing Key Laboratory of Urogenital Diseases (Male) Molecular Diagnosis and Treatment Center, Beijing 10034, China. [9] Department of General Surgery, Peking University First Hospital, Beijing 100034, China. [10] Key Laboratory of Carcinogenesis and Translational Research (Ministry of Education/ Beijing), Department of Nuclear Medicine; Peking University Cancer Hospital & Institute, Beijing 100142, China. [11] Translational Cancer Research Center, Peking University First Hospital, Beijing 100034, China. [12] Institute of Medical Technology, Peking University Health Science Center, Beijing, 100191, China.

*Corresponding authors.

E-mail address: yangxing2017@bjmu.edu.cn; cyril.barinka@ibt.cas.cz; yiguang.wang@pku.edu.cn.

†These authors contributed equally to this work.

Table of contents

1. Supplementary Figures

Supplementary Fig. 1. Synthesis route and characterization of 5, 6, Glu-490, ODAP-490 and ODAP-436. 3

Supplementary Fig. 2. Synthesis route and characterization of ODAP-490-COOH. 5

Supplementary Fig. 3. NMR analysis of compound 5, 6, ODAP-436, ODAP-490, Glu-490 and ODAP-490-COOH 6

Supplementary Fig. 4. Water solubility difference between Lys-Urea-Glu and Lys-Urea-ODAP scaffolds. 12

Supplementary Fig. 5. Functional characteristics of ODAP-490-COOH. 13

Supplementary Fig. 6. Structural characterization of the PSMA/Glu-490 complex. 14

Supplementary Fig. 7. Cytotoxicity comparison of ZJ-43, ODAP-FITC and ODAP-490 determined by the MTT viability assay. 15

Supplementary Fig. 8. Wash-free imaging of LNCaP and PC3 using Glu-490 and ODAP-490. 16

Supplementary Fig. 9. Inhibition of PSMA mediated endocytosis using different endocytosis inhibitors. 17

Supplementary Fig. 10. In vitro stability of ODAP-490. 18

Supplementary Fig. 11. Penetration depth of ODAP-490. 19

Supplementary Fig. 12. Bio-distribution of ODAP-490 and metabolites. 20

Supplementary Fig. 13. In vivo imaging in mouse model of prostate tumor with different doses of probes. 21

2. Supplementary note

Supplementary Note 1. PDB Validation Report 22

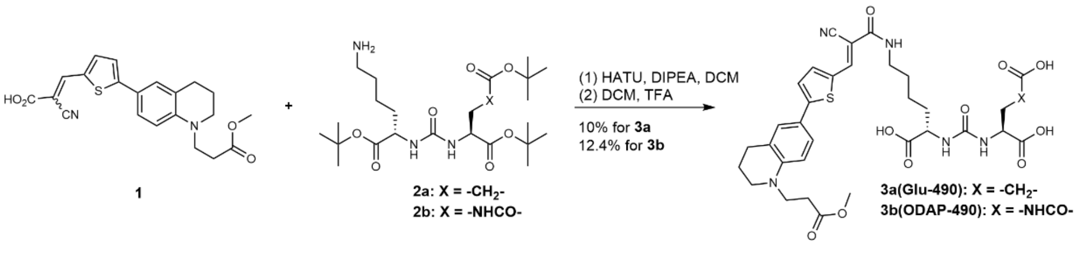

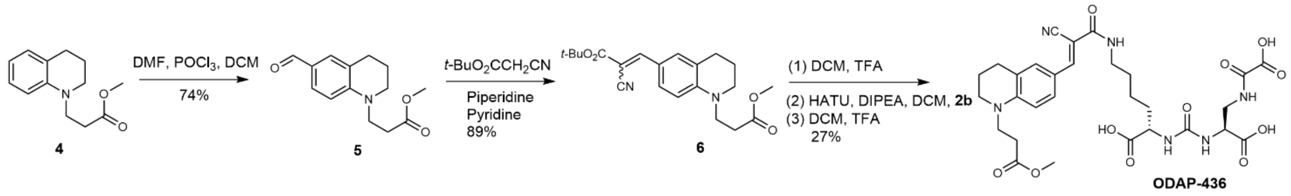

Supplementary Figure 1. Synthesis route and characterization of 5, 6 Glu-490, ODAP-490 and ODAP-436.

Glu-490 ^{1}H NMR (400 MHz, DMSO-d_6): δ 8.27 (s, 1H), 8.24 (t, J = 5.5 Hz, 1H), 7.79 (d, J = 4.1 Hz, 1H), 7.46 (d, J = 4.0 Hz, 1H), 7.42 (d, J = 8.9 Hz, 1H), 7.31 (s, 1H), 6.66 (d, J = 8.7 Hz, 1H), 6.35 – 6.28 (m, 2H), 4.14 – 4.02 (m, 2H), 3.65 – 3.57 (m, 5H), 3.31 – 3.29 (m, 2H), 3.21 – 3.14 (m, 2H), 2.76 – 2.68 (m, 2H), 2.63 – 2.54 (m, 2H), 2.29 – 2.19 (m, 2H), 1.97 – 1.79 (m, 3H), 1.76 – 1.60 (m, 2H), 1.59 – 1.44 (m, 3H), 1.35 – 1.25 (m, 2H); ^{13}C NMR (151 MHz, DMSO-d_6): δ 174.51, 174.14, 173.70, 172.05, 161.14, 157.29, 153.62, 145.71, 143.18, 140.34, 132.03, 126.72, 125.46, 122.66, 121.72, 119.57, 117.19, 110.57, 98.50, 52.29, 51.66, 51.43, 48.73, 46.42, 31.77, 30.68, 29.90, 28.69, 27.54, 27.30, 22.58, 21.29; HRMS (m/z): [M+H]$^+$ calcd. for [$C_{33}H_{39}N_5O_{10}S$]H$^+$, 698.2490; found, 698.2494.

ODAP-490 ^{1}H NMR (600 MHz, DMSO-d_6): δ 8.77 (t, J = 6.0 Hz, 1H), 8.26 (s, 1H), 8.23 (t, J = 5.6 Hz, 1H), 7.79 (d, J = 4.3 Hz, 1H), 7.45 (d, J = 4.0 Hz, 1H), 7.42 (dd, J = 8.6, 2.3 Hz, 1H), 7.31 (d, J = 2.3 Hz, 1H), 6.66 (d, J = 8.9 Hz, 1H), 6.51 (d, J = 7.9 Hz, 1H), 6.33 (d, J = 8.0 Hz, 1H), 4.27 (q, J = 6.8 Hz, 1H), 4.06 (dt, J = 12.9, 6.6 Hz, 1H), 3.63 – 3.58 (m, 5H), 3.41 (t, J = 6.7 Hz, 2H), 3.34 – 3.27 (m, 2H), 3.17 (q, J = 6.6 Hz, 2H), 2.73 (t, J = 6.2 Hz, 2H), 2.59 (t, J = 7.1 Hz, 2H), 1.84 (p, J = 6.3 Hz, 2H), 1.69 – 1.66 (m, 1H), 1.60 – 1.52 (m, 1H), 1.49 (dtd, J = 13.8, 9.5, 8.4, 4.4 Hz, 2H), 1.30 (p, J = 7.2 Hz, 2H); ^{13}C NMR (150 MHz, DMSO-d_6): δ 174.38, 172.59, 172.05, 161.65, 161.12, 158.26, 157.19, 153.60, 145.71, 143.18, 140.39, 132.01, 126.71, 125.46, 122.65, 121.72, 119.55, 117.17, 110.56, 98.48, 52.29, 52.15, 51.44, 48.72, 46.40, 40.88, 40.04, 31.79, 30.66, 28.68, 27.29, 22.56, 21.27. HRMS (m/z): [M+H]$^+$ calcd. for [$C_{33}H_{38}N_6O_{11}S$]H$^+$, 727.2392; found, 727.2396.

(5) ^{1}H NMR (400 MHz, Chloroform-d): δ 9.67 (s, 1H), 7.55 (dd, J = 8.6, 2.1 Hz, 1h), 7.47 (d, J = 4.0 Hz, 1H), 6.60 (d, J = 8.6 Hz, 1H), 3.75 – 3.64 (m, 5H), 3.45 – 3.38 (m, 2H), 2.78 (t, J = 6.3 Hz, 2H), 2.67 – 2.60 (m, 2H), 1.99 – 1.91 (m, 2H); ^{13}C NMR (101 MHz, Chloroform-d): δ 190.25, 172.25, 149.73, 131.22, 130.60, 125.31, 122.29, 109.43, 51.99, 49.89, 47.14, 31.32, 27.91, 21.56; HRMS (m/z): [M+H]$^+$ calcd. for [$C_{14}H_{17}NO_3$]H$^+$, 248.1281; found, 248.1278.

(6)^{1}H NMR (400 MHz, Chloroform-d): δ 7.84 (s, 1H), 7.66 (dd, J = 8.8, 2.2 Hz, 1H), 7.55 (d, J = 1.9 Hz, 1H), 6.52 (d, J = 8.9 Hz, 1H), 3.66 – 3.58 (m, 5H), 3.42 – 3.30 (m, 2H), 2.68 (t, J = 6.2 Hz, 2H), 2.57 (t, J = 7.1 Hz, 2H), 1.93 – 1.82 (m, 2H), 1.50 (s, 9H); ^{13}C NMR (101 MHz, Chloroform-d): δ 171.88, 163.09, 153.37, 148.83, 132.61, 132.53, 122.38, 119.42, 117.63, 109.86, 95.26, 82.20, 51.79, 49.78, 46.87, 31.22, 27.98, 27.70, 21.30; HRMS (m/z): [M+H]$^+$ calcd. for [$C_{21}H_{26}N_2O_4$]H$^+$, 371.1965; found, 371.1963.

ODAP-436 ^{1}H NMR (400 MHz, DMSO-d_6): δ 8.78 (t, J = 6.0 Hz, 1H), 8.06 (t, J = 5.6 Hz, 1H), 7.87 (s, 1H),

7.69 (dd, J = 8.9, 2.2 Hz, 1H), 7.57 (d, J = 2.0 Hz, 1H), 6.73 (d, J = 9.0 Hz, 1H), 6.51 (d, J = 8.0 Hz, 1H), 6.33 (d, J = 8.1 Hz, 1H), 4.27 (q, J = 6.6 Hz, 1H), 4.05 (td, J = 7.8, 5.5 Hz, 1H), 3.65 (t, J = 7.1 Hz, 2H), 3.60 (s, 3H), 3.42 – 3.36 (m, 4H), 3.21 – 3.08 (m, 2H), 2.68 (t, J = 6.1 Hz, 2H), 2.61 (t, J = 7.1 Hz, 2H), 1.84 (p, J = 6.4 Hz, 2H), 1.70 –1.62 (m, 1H), 1.59 – 1.41 (m, 3H), 1.32 – 1.25(m, 2H); ^{13}C NMR (101 MHz, DMSO-d_6) δ 174.96, 173.16, 172.43, 162.58, 162.21, 158.81, 157.74, 150.59, 148.87, 132.06, 131.95, 122.57, 119.27, 118.79, 110.70, 97.04, 52.85, 52.70, 52.02, 49.50, 46.91, 41.41, 41.40, 32.36, 31.26, 29.32, 27.83, 23.14, 21.45; HRMS (m/z): $[M+H]^+$ calcd. for $[C_{29}H_{36}N_6O_{11}]H^+$, 645.2514; found, 645.2513.

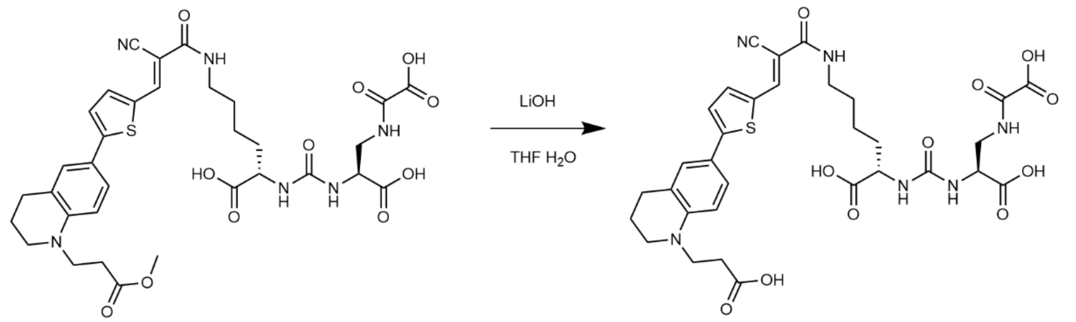

Supplementary Figure 2. Synthesis route and characterization of ODAP-490-COOH.

(4S,8S)-16-(5-(1-(2-carboxyethyl)-1,2,3,4-tetrahydroquinolin-6-yl)thiophen-2-yl)-15-cyano-1,6,14-trioxo-2,5,7,13-tetraazahexadec-15-ene-1,4,8-tricarboxylic acid

ODAP-490 (12 mg, 0.017 mmol) was dissolved in THF/H2O (v/v=1:1, 2mL), then LiOH (7mg, 0.17 mmol) was added and the reaction mixture was stirred for 15min at room temperature. Then, the mixture was neutralized to PH = 7 with 1M HCl and concentrated under reduced pressure. The crude product was purified by HPLC (0-5 min, 5% MeCN (0.1%TFA); 5-15 min, 10%-45% MeCN (0.1%TFA); 15-25 min, 45% MeCN (0.1%TFA); 25-26 min, 45%-90% MeCN (0.1%TFA); 26-31 min, 90% MeCN (0.1%TFA) to yield as a red solid (3mg, yield 22%)).

ODAP-490-COOH ^{1}H NMR (600 MHz, DMSO-d_6): δ 8.72 (t, J = 6.0 Hz, 1H), 8.23 (s, 1H), 8.18 (t, J = 5.6 Hz, 1H), 7.75 (d, J = 3.9 Hz, 1H), 7.42 (d, J = 4.0 Hz, 1H), 7.39 (dd, J = 8.6, 2.3 Hz, 1H), 7.27 (d, J = 2.3 Hz, 1H), 6.63 (d, J = 8.9 Hz, 1H), 6.47 (d, J = 8.1 Hz, 1H), 6.29 (d, J = 8.1 Hz, 2H), 4.27 – 4.21 (m, 1H), 4.02 (td, J = 7.9, 5.3 Hz, 1H), 3.54 (t, J = 7.1 Hz, 2H), 3.37 (t, J = 6.8 Hz, 2H), 3.14 (q, J = 6.9 Hz, 2H), 2.69 (t, J = 6.2 Hz, 2H), 1.81 (p, J = 6.3 Hz, 2H), 1.66 – 1.59 (m, 1H), 1.55 – 1.40 (m, 3H), 1.29 – 1.23 (m, 1H); ^{13}C NMR (101 MHz, DMSO-d_6) δ 174.43, 173.21, 172.63, 161.71, 161.15, 158.44, 157.23, 153.70, 145.83, 143.21, 140.44, 131.98, 126.70, 125.51, 122.62, 121.69, 119.45, 117.22, 110.57, 98.43, 52.32, 52.19, 48.74, 46.49, 40.90, 31.81, 30.95, 28.71, 27.33, 22.59, 21.31; HRMS (m/z): $[M+H]^+$ calcd. for $[C_{32}H_{36}N_6O_{11}S]H^+$, 713.2248; found, 713.2238.

Supplementary Figure 3. NMR analysis of compound 5, 6, ODAP-436, ODAP-490, Glu-490 and ODAP-490-COOH

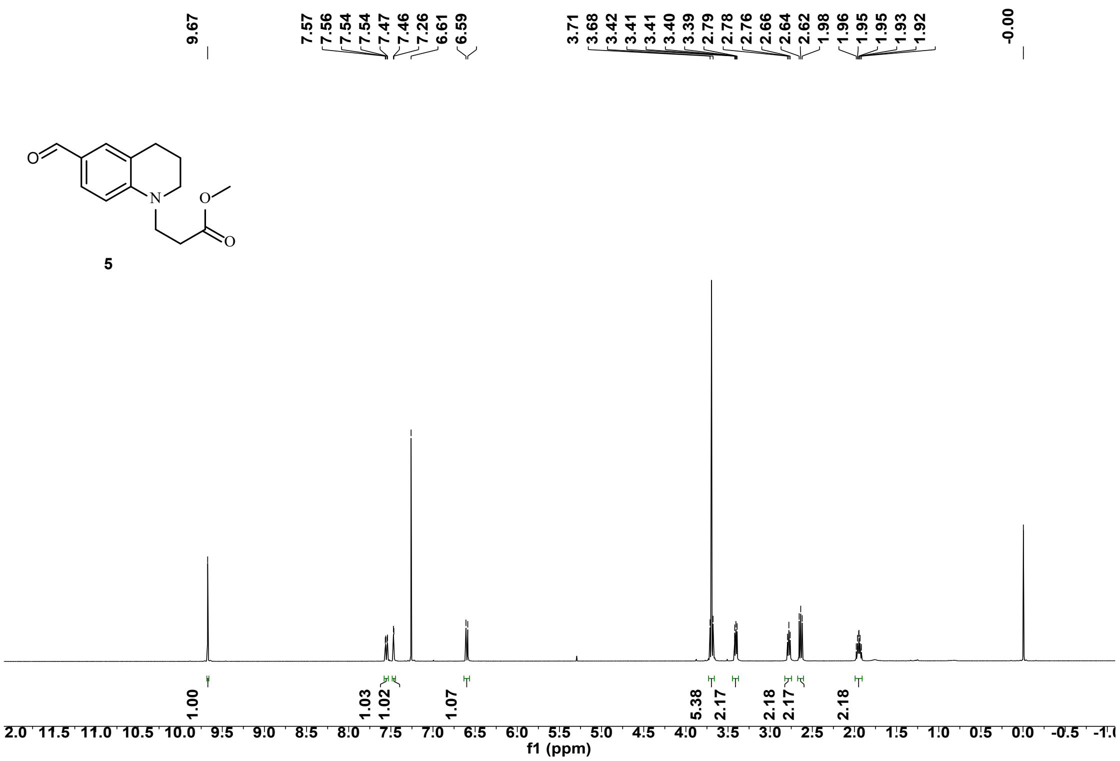

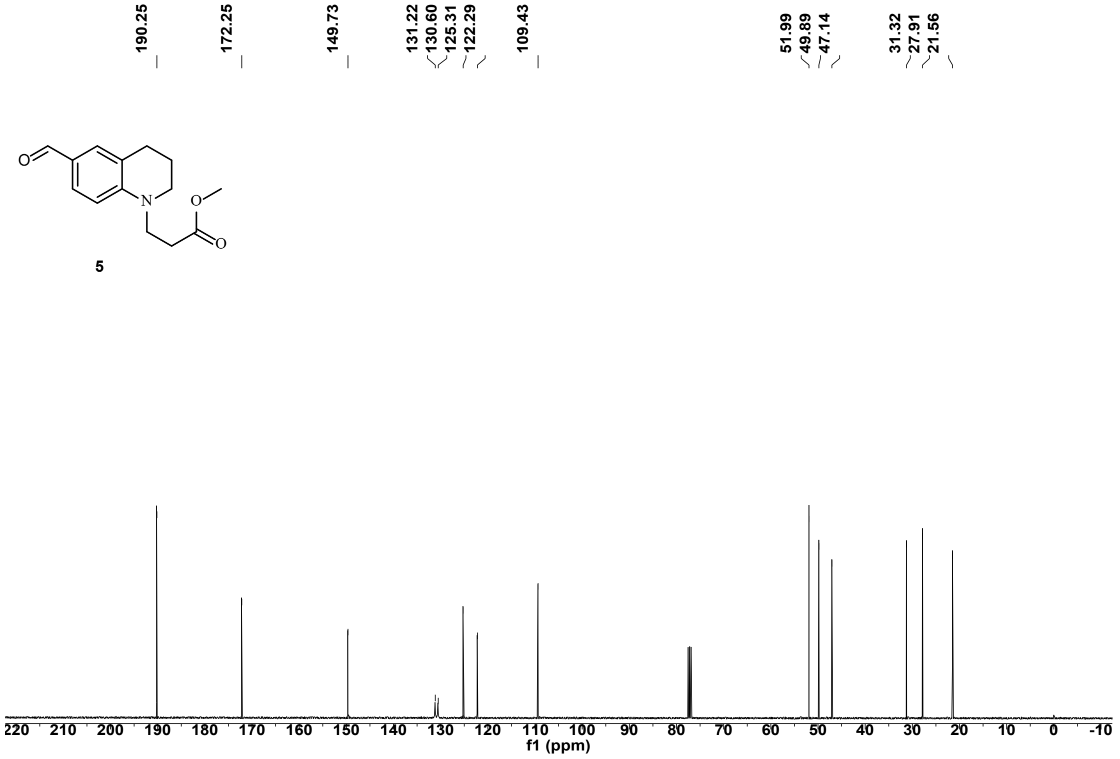

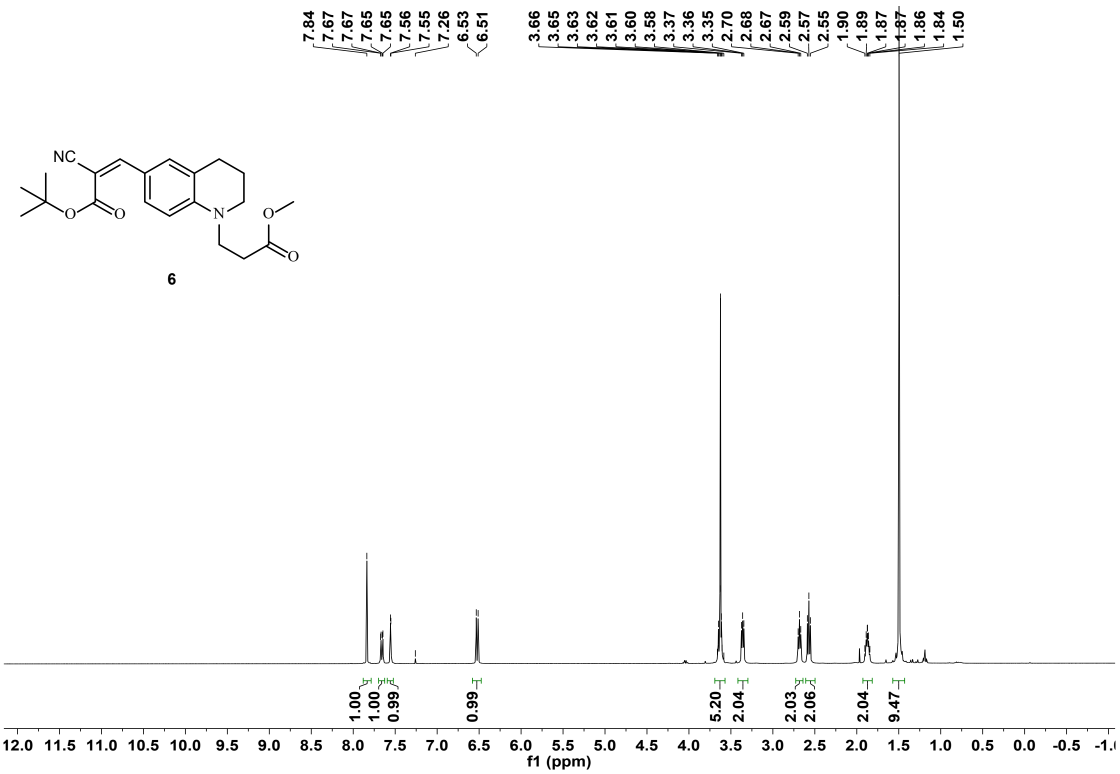
7.84 7.67 7.67 7.65 7.65 7.56 7.55 7.26 6.53 6.51
3.66 3.65 3.63 3.62 3.61 3.60 3.58 3.37 3.36 3.35 2.70 2.68 2.67 2.59 2.57 2.55 1.90 1.89 1.87 1.87 1.86 1.84 1.50
NC
O
N
1.00 1.00 0.99 0.99 5.20 2.04 2.03 2.06 2.04 9.47
f1 (ppm)

6

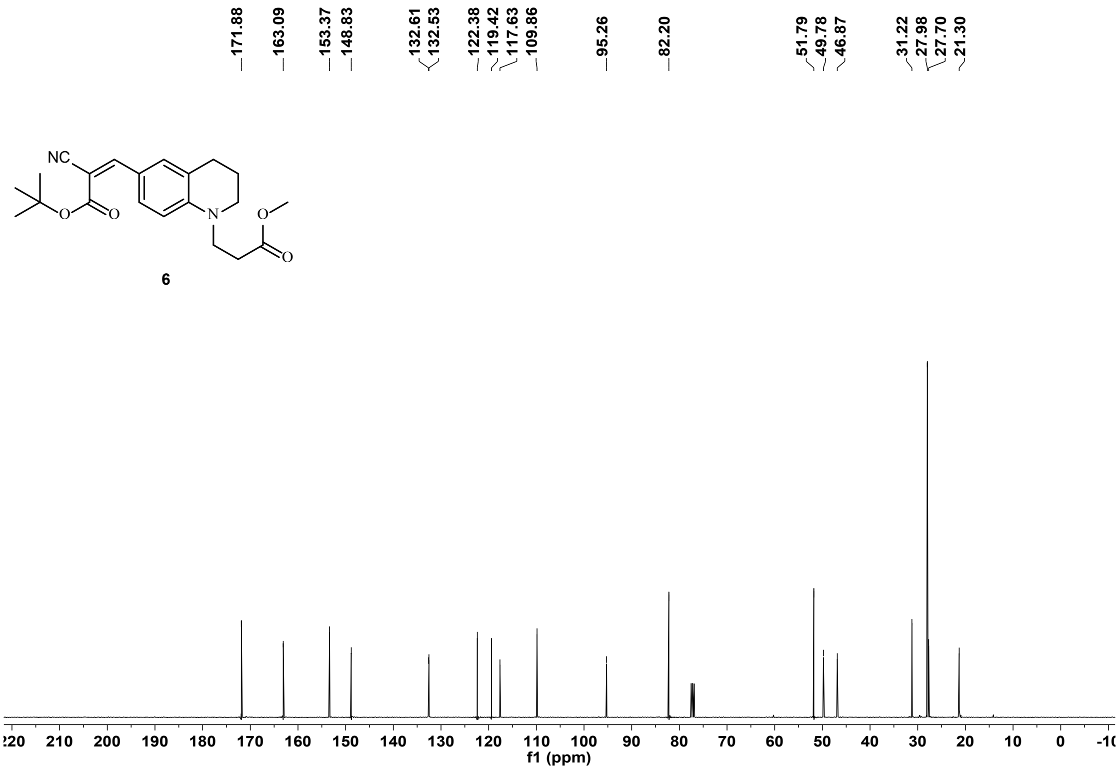
171.88 163.09 153.37 148.83 132.61 132.53 122.38 119.42 117.63 109.86 95.26 82.20 51.79 49.78 46.87 31.22 27.98 27.70 21.30
NC
O
N
f1 (ppm)

6

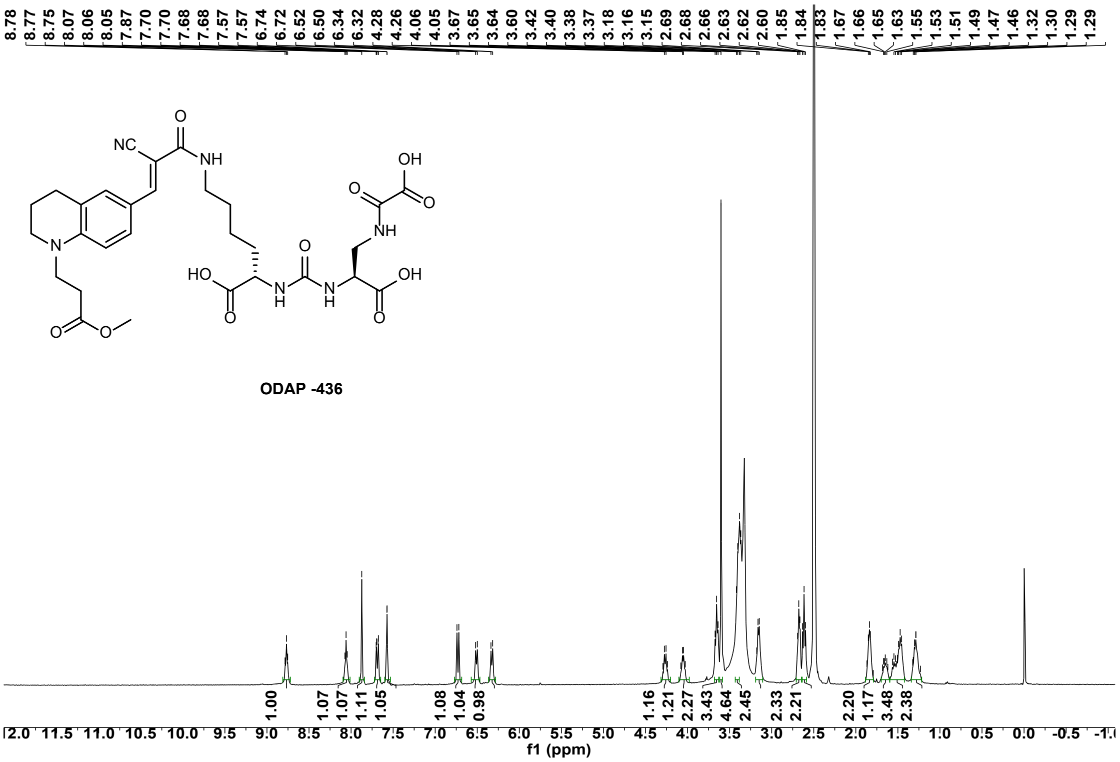

8.78 8.77 8.75 8.07 8.06 8.05 7.87 7.70 7.70 7.68 7.57 7.57 6.74 6.72 6.52 6.50 6.34 6.32 4.28 4.26 4.06 4.05 3.67 3.65 3.64 3.60 3.42 3.40 3.38 3.37 3.18 3.16 3.15 2.69 2.68 2.66 2.63 2.62 2.60 1.85 1.84 1.83 1.67 1.66 1.65 1.63 1.55 1.53 1.51 1.49 1.47 1.46 1.32 1.30 1.29 1.29
ODAP -436
1.00 1.07 1.07 1.11 1.05 1.08 1.04 0.98 1.16 1.21 2.27 3.43 4.64 2.45 2.33 2.21 2.20 1.17 3.48 2.38
f1 (ppm)

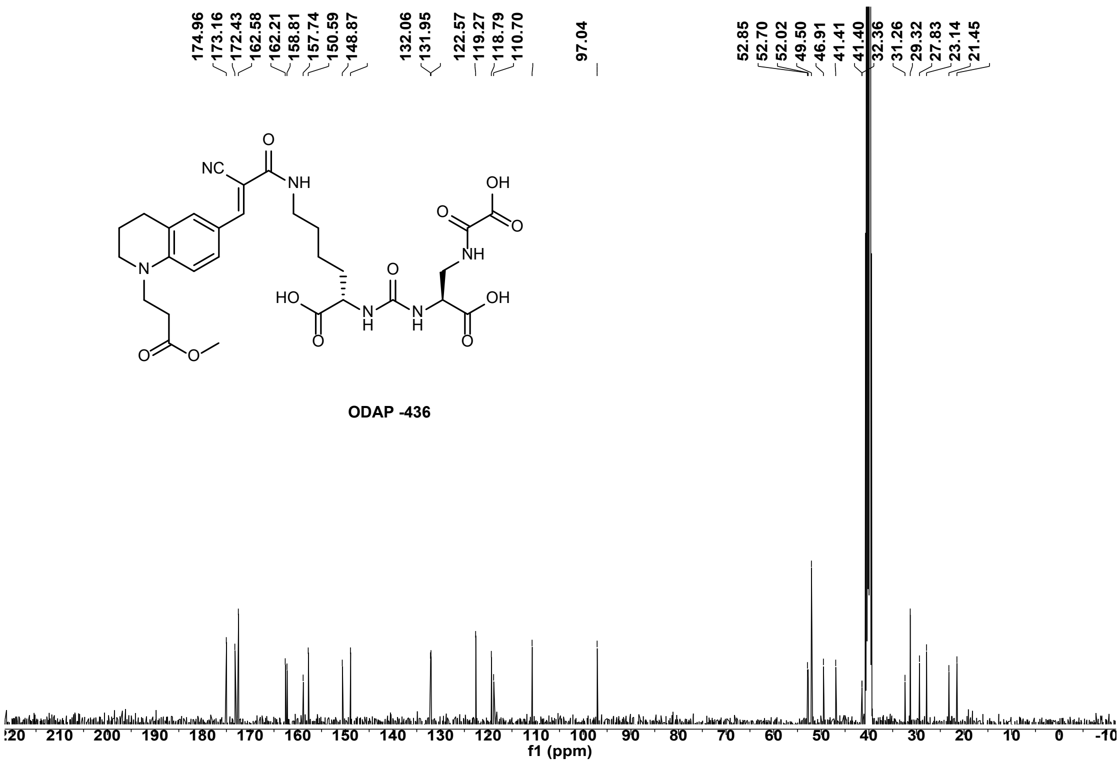

174.96 173.16 172.43 162.58 162.21 158.81 157.74 150.59 148.87 132.06 131.95 122.57 119.27 118.79 110.70 97.04 52.85 52.70 52.02 49.50 46.91 41.41 41.40 32.36 31.26 29.32 27.83 23.14 21.45
ODAP -436
f1 (ppm)

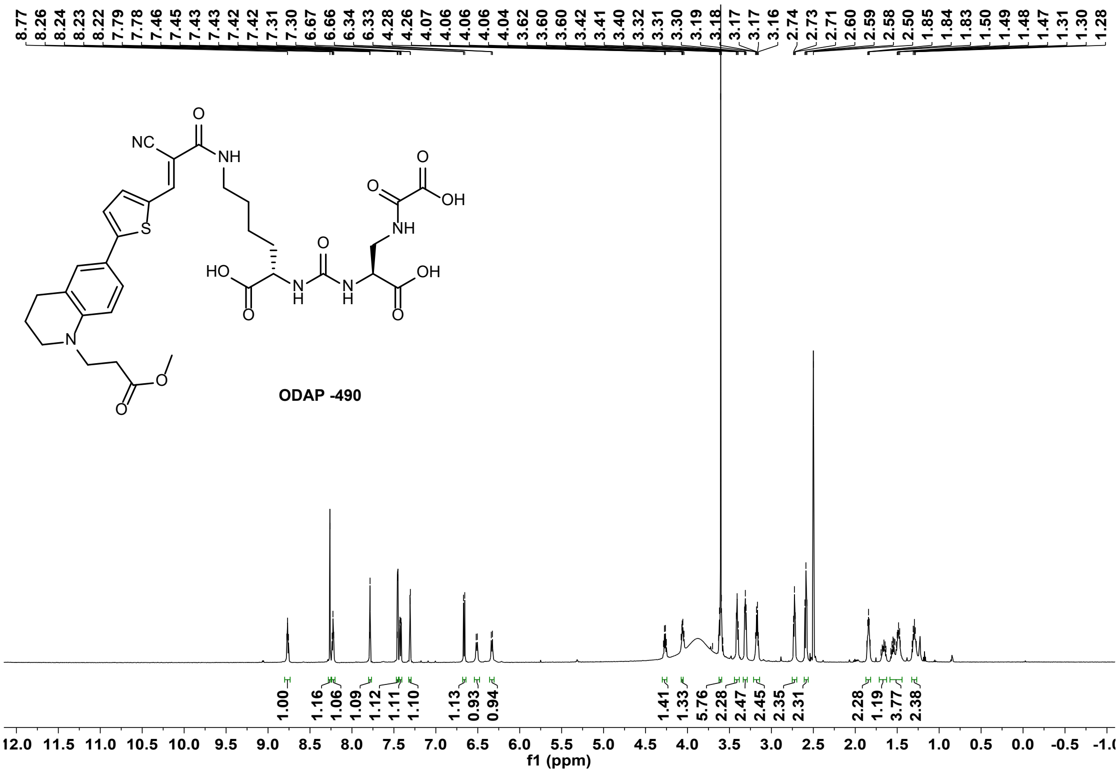
8.77 8.26 8.24 8.23 8.22 7.79 7.78 7.46 7.45 7.43 7.43 7.42 7.42 7.31 7.30 6.67 6.66 6.34 6.33 4.28 4.26 4.07 4.06 4.06 4.04 3.62 3.60 3.60 3.42 3.41 3.40 3.32 3.31 3.30 3.19 3.18 3.17 3.17 3.16 2.74 2.73 2.71 2.60 2.59 2.58 2.50 1.85 1.84 1.83 1.50 1.49 1.48 1.47 1.31 1.30 1.28
ODAP -490
1.00 1.16 1.06 1.09 1.12 1.11 1.10 1.13 0.93 0.94 1.41 1.33 5.76 2.28 2.47 2.45 2.35 2.31 2.28 1.19 3.77 2.38
12.0 11.5 11.0 10.5 10.0 9.5 9.0 8.5 8.0 7.5 7.0 6.5 6.0 5.5 5.0 4.5 4.0 3.5 3.0 2.5 2.0 1.5 1.0 0.5 0.0 -0.5 -1.(
f1 (ppm)

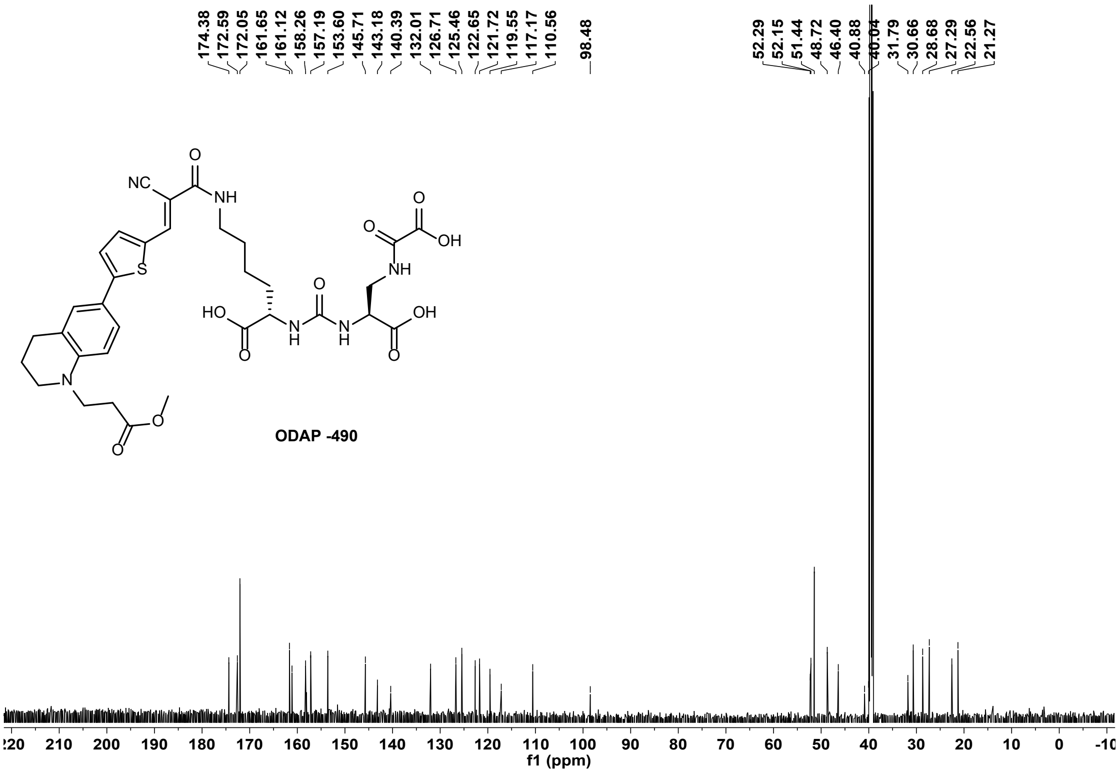
174.38 172.59 172.05 161.65 161.12 158.26 157.19 153.60 145.71 143.18 140.39 132.01 126.71 125.46 122.65 121.72 119.55 117.17 110.56 98.48
52.29 52.15 51.44 48.72 46.40 40.88 40.04 31.79 30.66 28.68 27.29 22.56 21.27
ODAP -490
220 210 200 190 180 170 160 150 140 130 120 110 100 90 80 70 60 50 40 30 20 10 0 -1(
f1 (ppm)

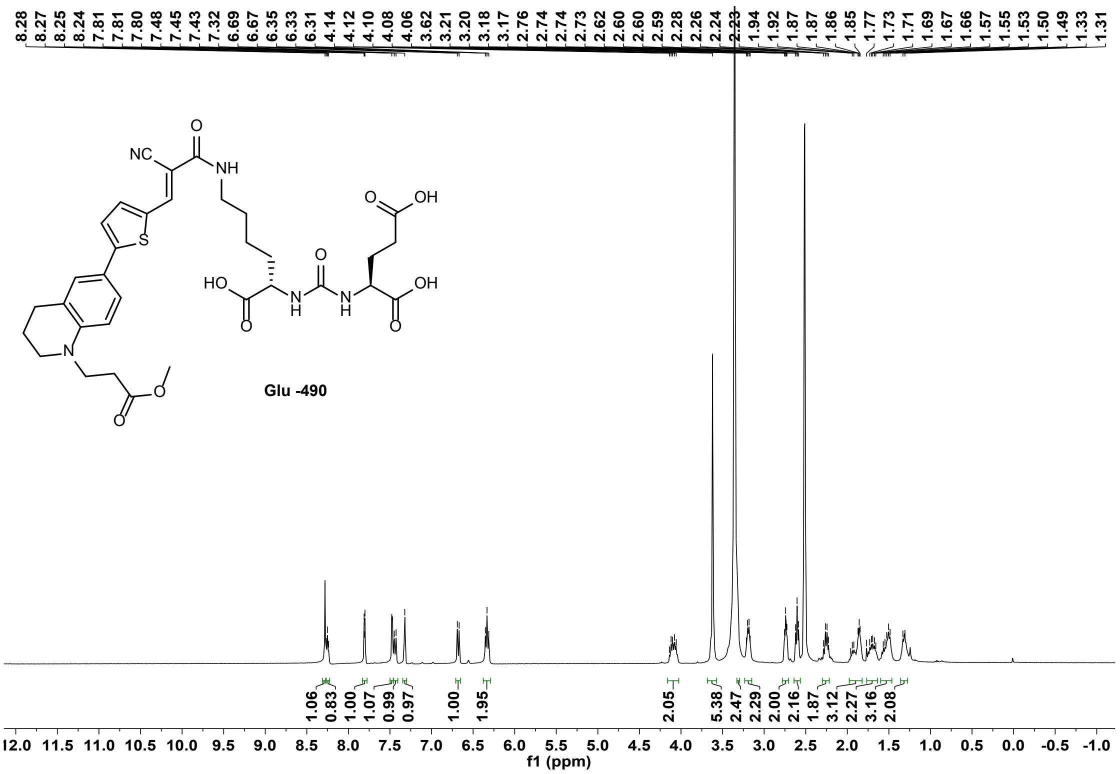
8.28 8.27 8.25 8.24 7.81 7.81 7.80 7.48 7.45 7.43 7.32 6.69 6.67 6.35 6.33 6.31 4.14 4.12 4.10 4.08 4.06 3.62 3.21 3.20 3.18 3.17 2.76 2.74 2.74 2.73 2.62 2.60 2.60 2.59 2.28 2.26 2.24 2.23 1.94 1.92 1.87 1.87 1.86 1.85 1.77 1.73 1.71 1.69 1.67 1.66 1.57 1.55 1.53 1.50 1.49 1.33 1.31
NC
O
NH
O
OH
S
O
HO
N
H
N
H
OH
O
O
N
O
O
Glu -490
1.06 0.83 1.00 1.07 0.99 0.97 1.00 1.95 2.05 5.38 2.47 2.29 2.00 2.16 1.87 3.12 2.27 3.16 2.08
12.0 11.5 11.0 10.5 10.0 9.5 9.0 8.5 8.0 7.5 7.0 6.5 6.0 5.5 5.0 4.5 4.0 3.5 3.0 2.5 2.0 1.5 1.0 0.5 0.0 -0.5 -1.0
f1 (ppm)

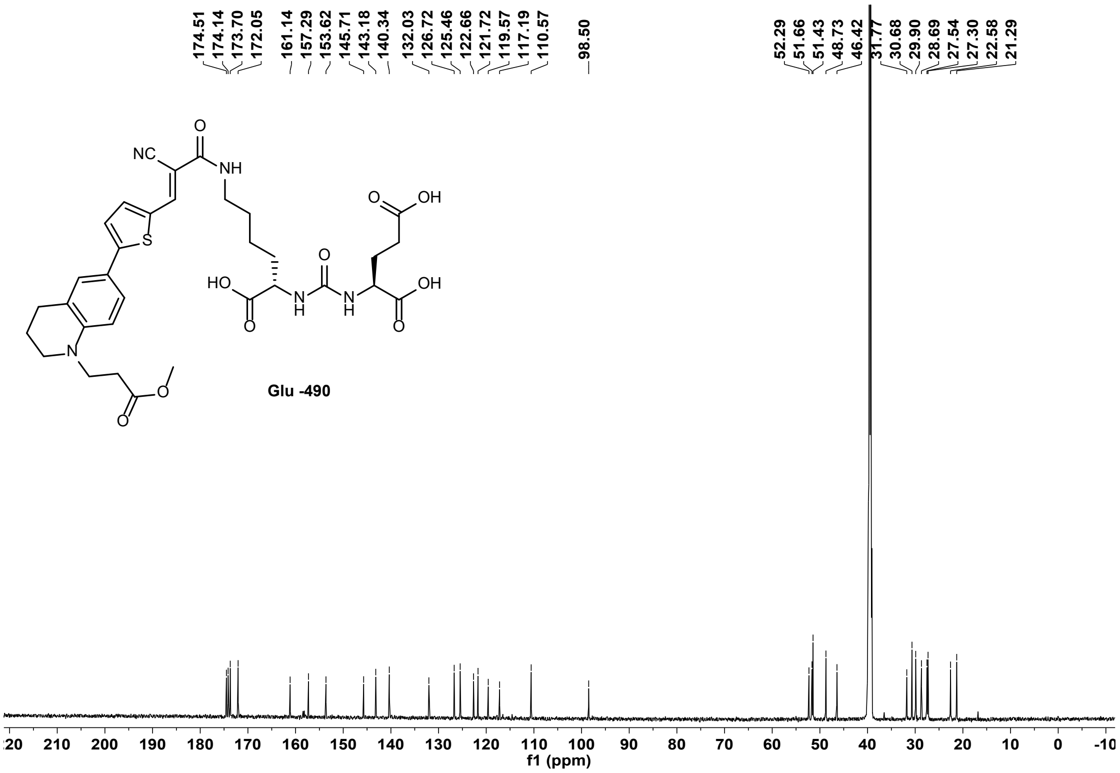
174.51 174.14 173.70 172.05 161.14 157.29 153.62 145.71 143.18 140.34 132.03 126.72 125.46 122.66 121.72 119.57 117.19 110.57 98.50
52.29 51.66 51.43 48.73 46.42 31.77 30.68 29.90 28.69 27.54 27.30 22.58 21.29
NC
O
NH
O
OH
S
O
HO
N
H
N
H
OH
O
O
N
O
O
Glu -490
220 210 200 190 180 170 160 150 140 130 120 110 100 90 80 70 60 50 40 30 20 10 0 -10
f1 (ppm)

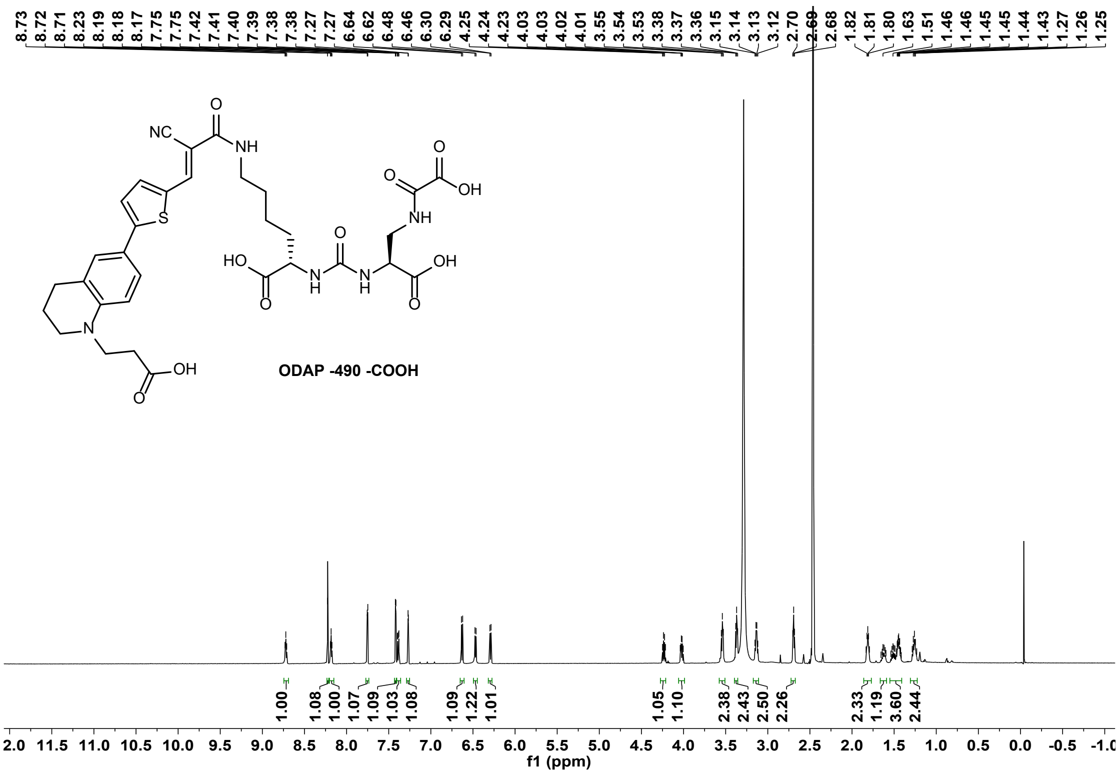
8.73 8.72 8.71 8.23 8.19 8.18 8.17 7.75 7.75 7.42 7.41 7.40 7.39 7.38 7.38 7.27 7.27 6.64 6.62 6.48 6.46 6.30 6.29 4.25 4.24 4.23 4.03 4.03 4.02 4.01 3.55 3.54 3.53 3.38 3.37 3.36 3.15 3.14 3.13 3.12 2.70 2.69 2.68 1.82 1.81 1.80 1.63 1.51 1.46 1.45 1.45 1.44 1.43 1.27 1.26 1.25
NC
O
NH
S
N
OH
O
HO
H
N
H
N
OH
NH
ODAP -490 -COOH
1.00 1.08 1.00 1.07 1.09 1.03 1.08 1.09 1.22 1.01 1.05 1.10 2.38 2.43 2.50 2.26 2.33 1.19 3.60 2.44
f1 (ppm)

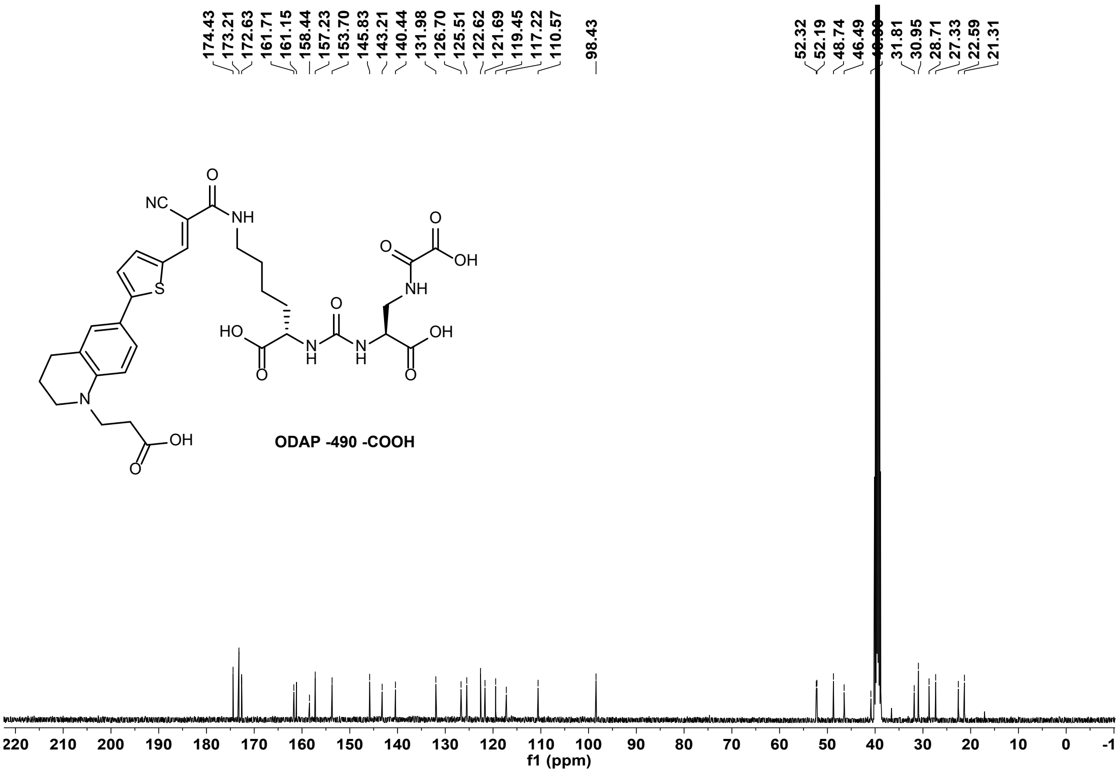
174.43 173.21 172.63 161.71 161.15 158.44 157.23 153.70 145.83 143.21 140.44 131.98 126.70 125.51 122.62 121.69 119.45 117.22 110.57 98.43
52.32 52.19 48.74 46.49 31.81 30.95 28.71 27.33 22.59 21.31
ODAP -490 -COOH
f1 (ppm)

a

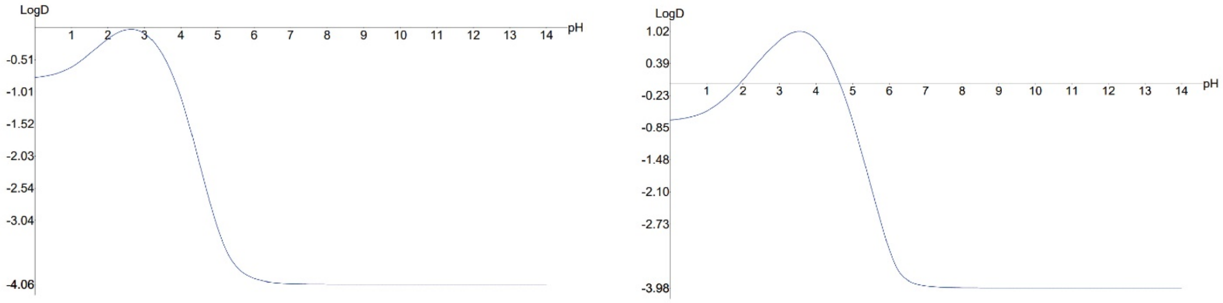

ODAP-490 Glu-490

b

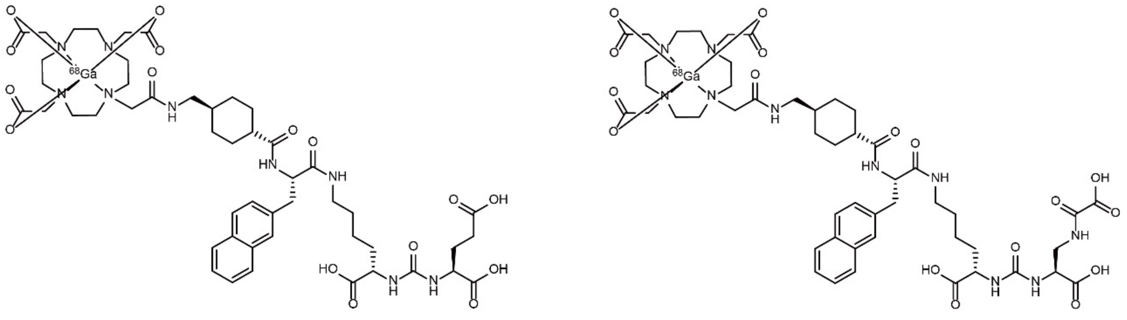

^{68}Ga-PSMA617 ^{68}Ga-**7**

Supplementary Figure 4. Water solubility difference between Lys-Urea-Glu and Lys-Urea-ODAP scaffolds. (**a**) The log D values of ODAP-490 and Glu-490 calculated by ACD/Labs. (**b**) Structures of ^{68}Ga-PSMA617 and ^{68}Ga-**7,** with the experimental log *P* values of -2.00 ± 0.27 and -2.62 ± 0.30, respectively.

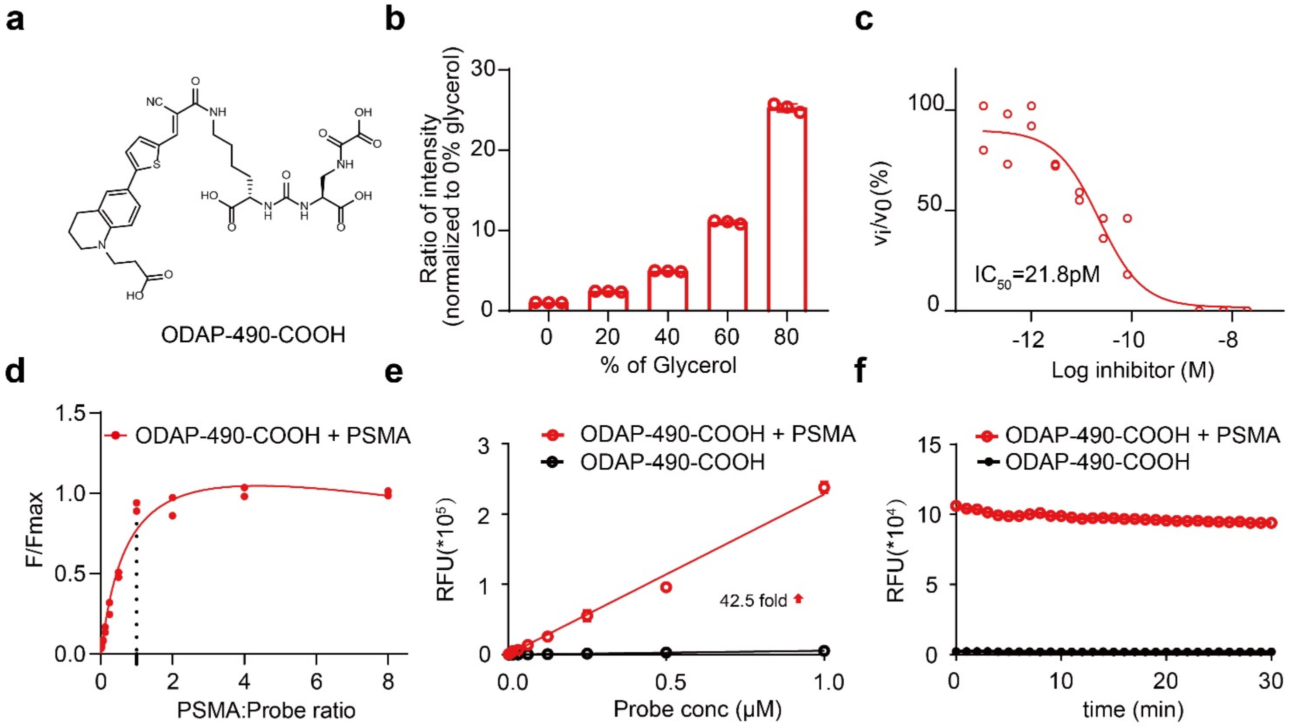

Supplementary Figure 5. Functional characteristics of ODAP-490-COOH. (**a**) Structure of ODAP-490-COOH. (**b**) Changes in fluorescence intensity of ODAP-490-COOH in solutions with increasing glycerol concentrations. Probe concentration was 0.01 mM and data were normalized to PBS control. Data are shown as mean ± s.d. ($n = 3$ biologically independent experiments) (**c**) Inhibition of PSMA enzymatic activity using the radioenzymatic assay. ($n = 2$ biologically independent experiments) (**d**) Saturation binding of rhPSMA/probe complexes. ($n = 2$ biologically independent experiments) (**e**) Fluorescence intensity of the rhPSMA/probe complex in response to concentration changes. ($n = 2$ biologically independent experiments). RFU: relative fluorescence units. (**f**) Time frame for rhPSMA/probe complex formation. Data are shown as mean ± s.d. ($n = 3$ biologically independent experiments). RFU: relative fluorescence units. Source data are provided in Source Data file.

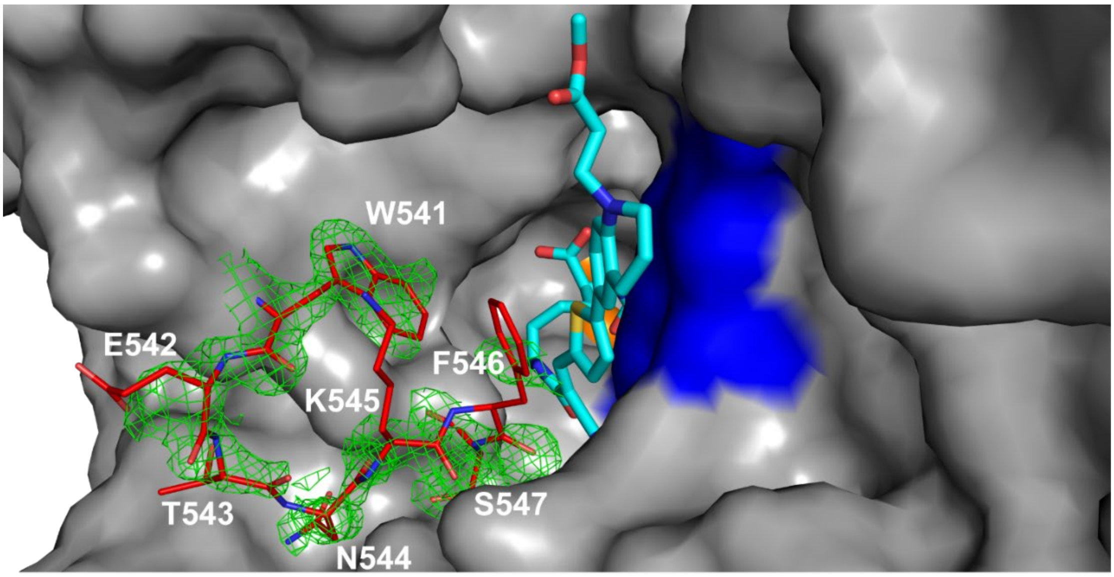

Supplementary Figure 6. Structural characterization of the PSMA/Glu-490 complex. PSMA is shown in surface representation (gray) with residues of the glutarate sensor interaction with the FMR moiety colored blue. Residues comprising the entrance lid (W541 – S547) are shown as lines with caron atoms colored red. The *Fo-Fc* omit map (green) is contoured at 3.0 σ and the inhibitor is shown in stick representation with carbon atoms colored cyan, PDB code (7BFZ).

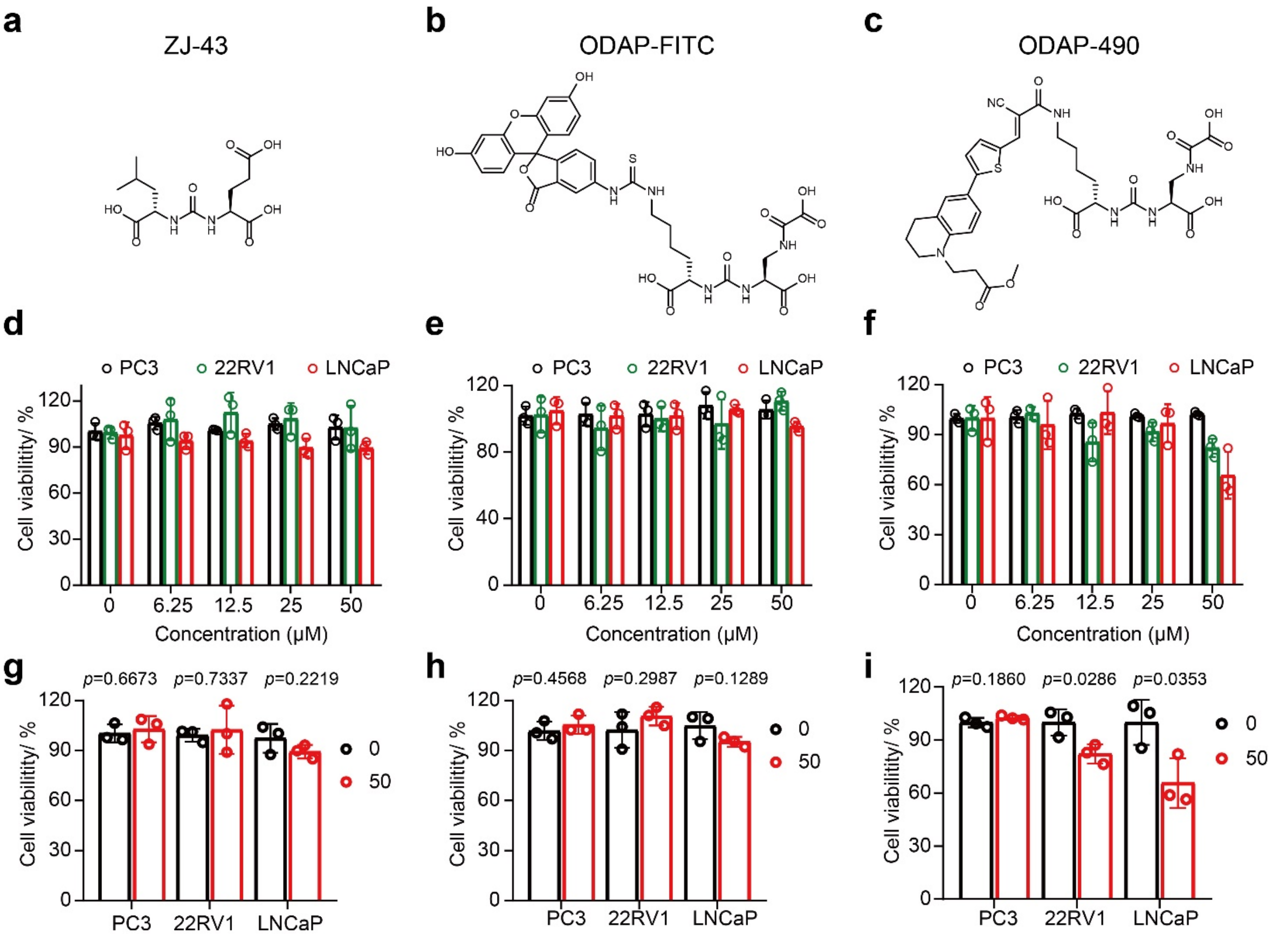

Supplementary Figure 7, Cytotoxicity comparison of ZJ-43, ODAP-FITC and ODAP-490 determined by the MTT viability assay. (a-c) Structures of ZJ-43, ODAP-FITC and ODAP-490. **(d-f)** Cytotoxicity of ZJ-43, ODAP-FITC and ODAP-490 at different probe concentrations. (*n*=3 independent experiments). **(g-i)** Comparison of cytotoxicity between 0 μM and 50 μM for each compound. Data are shown as mean ± s.d. (*n*=3 independent experiments). Two-tailed unpaired Student t-test. Source data are provided in Source Data file.

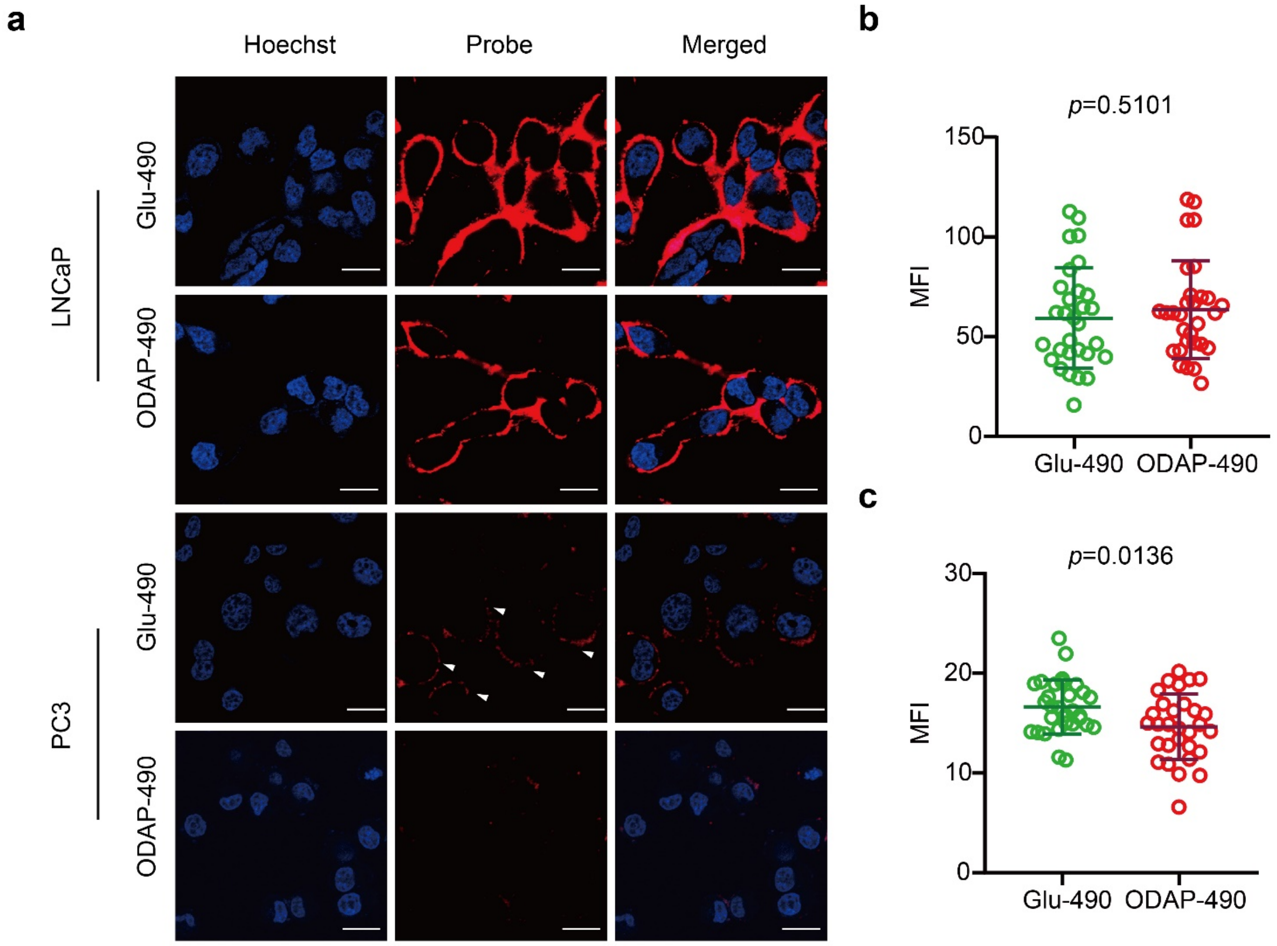

Supplementary Figure 8, Wash-free imaging of LNCaP and PC3 using Glu-490 and ODAP-490. (**a**) Confocal imaging of LNCaP and PC3 stained with Glu-490 and ODAP-490. White arrows indicate the nonspecific staining. Scale bar: 20 μm. (**b**) Quantification of the fluorescence intensity of LNCaP cells in panel **a**, *(n* = 30 biologically independent cell samples). (**c**) Quantification of the fluorescence intensity of PC3 cells in panel **a**, *(n* = 30 biologically independent cell samples). MFI: mean fluorescence intensity. Two-tailed unpaired Student t-test. Source data are provided in Source Data file.

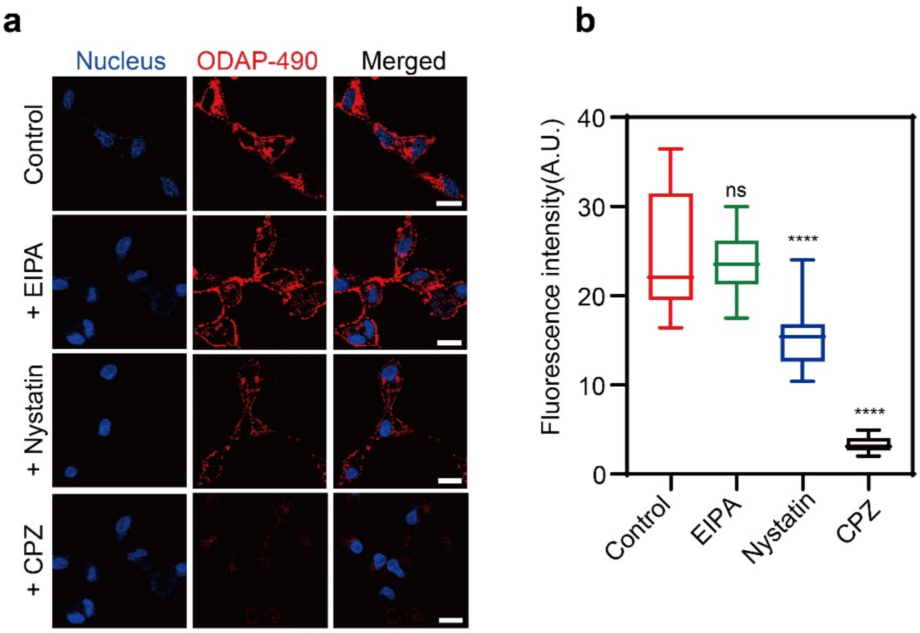

Supplementary Figure 9. Inhibition of PSMA mediated endocytosis using different endocytosis inhibitors. **(a)** LNCaP cells were treated with 10 μM **ODAP-490** for 2 hours, with or without preincubated with 10 μM endocytosis inhibitors for 30 minutes. The fluorescence images were captured by a confocal microscope. Scale bar: 20 μm. **(b)** Quantification of the fluorescence intensity of samples in panel **a**, Upper and lower bounds of boxes represent 25th and 75th percentiles, horizontal lines indicate the median values, whiskers represent the minimum and maximum ranges. (*n*=20 biologically independent cells). ****$P < 0.0001$, P values = 0.61(EIPA); 1.09e^{-5}(Nystatin); 1.14e^{-11}(CPZ); two-tailed unpaired student t-test. CPZ: Chlorpromazine; Source data are provided in Source Data file.

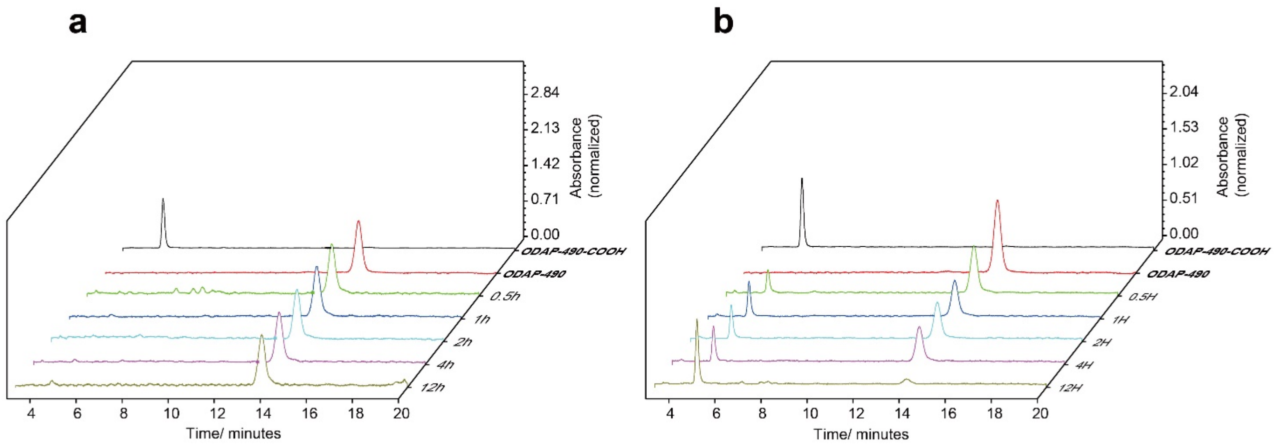

Supplementary Figure 10. In vitro stability of ODAP-490. (**a**) HPLC spectra of ODAP-490 incubated with human serum albumin (HSA) for different times. (**b**) HPLC spectra of ODAP-490 incubated with murine serum for different times. Source data are provided in Source Data file.

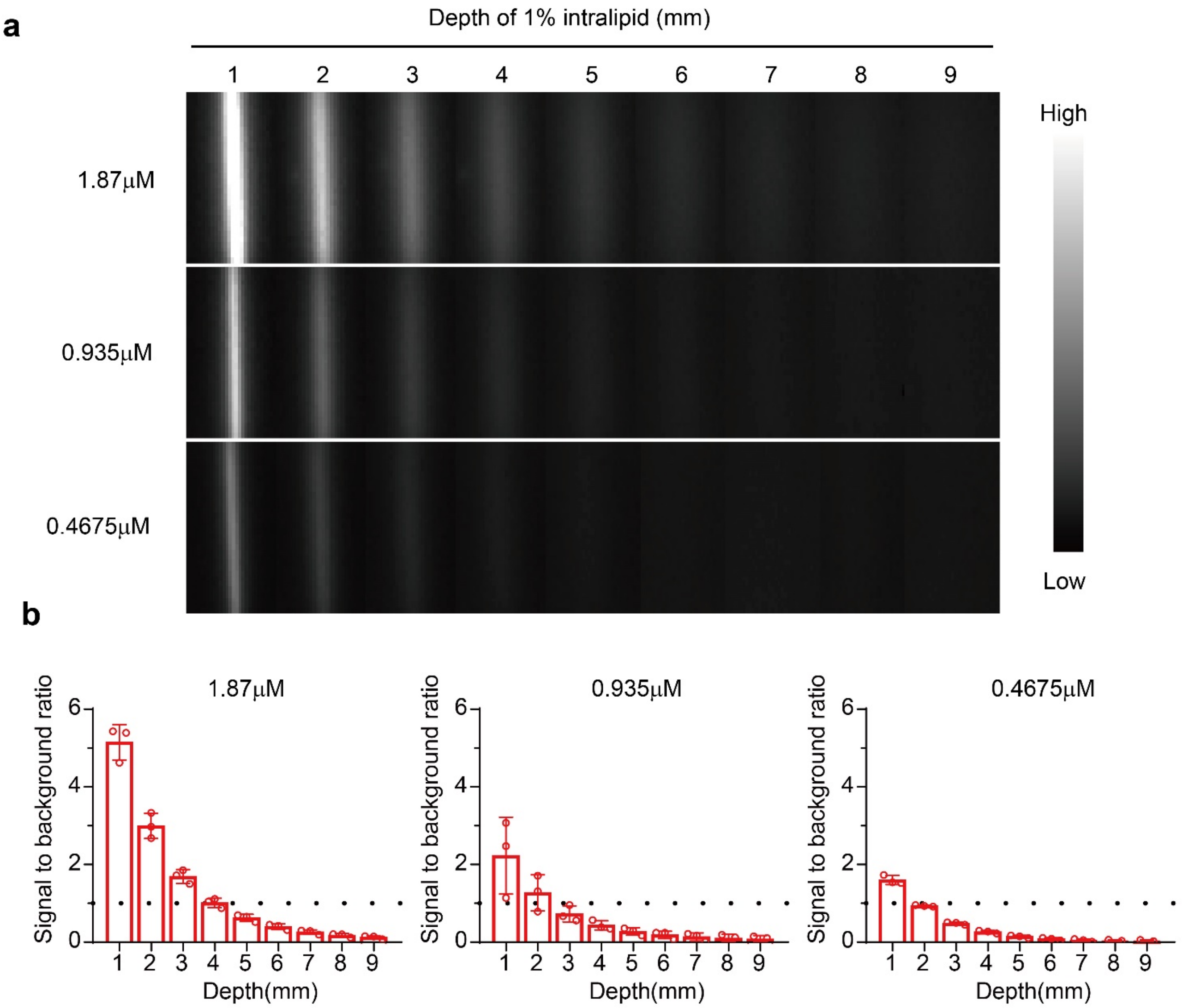

Supplementary Figure 11. Penetration depth of ODAP-490. (**a**) Fluorescence images of ODAP-490 immersed in different depth of 1% intralipid. (**b**)Signal to background ratio in panel **a.** The background signal was subtracted from the signal of the region of interest. Data are shown as mean ± s.d. *(n* = 3 biologically independent experiments). Source data are provided in Source Data file.

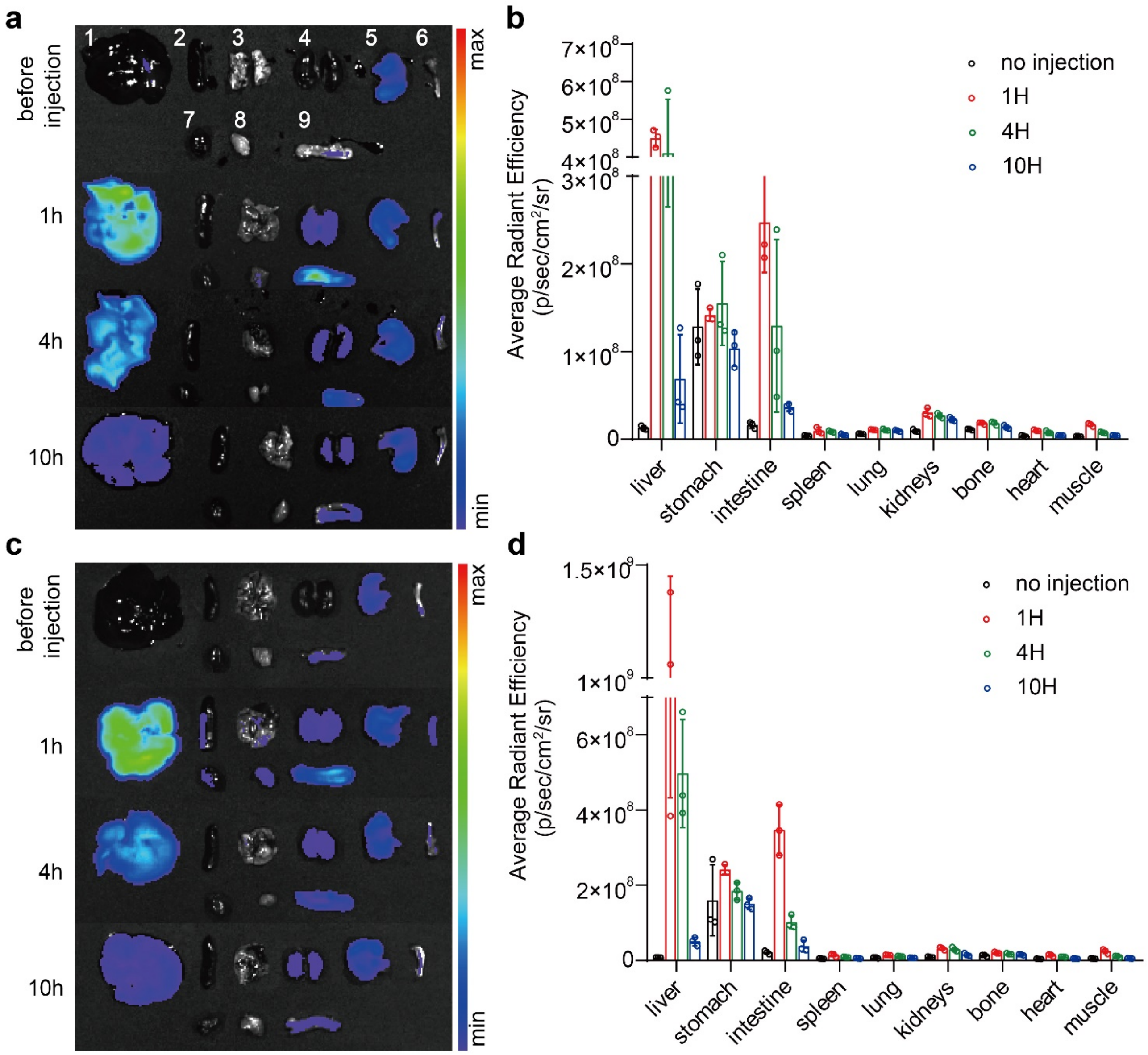

Supplementary Figure 12. Bio-distribution of ODAP-490 and metabolites. (a) The image of the excised organs before injection and 1 h, 4 h, and 10 h post-injection of 25 nmol ODAP-490. The organs sampled include: (1) liver, (2) spleen, (3) lung, (4) kidneys, (5) stomach, (6) bone, (7) heart, (8) muscle, (9) intestine. **(b)** Quantification of the fluorescence intensity of organs in panel a, Data are shown as mean ± s.d. *(n* = 3 biologically independent mice for each time point). **(c)** Image of the excised organs before injection and 1 h, 4 h, and 10 h post-injection of 25 nmol hydrolyzed ODAP-490. **(d)** Quantification of the fluorescence intensity of organs in panel c, Data are shown as mean ± s.d. *(n* = 3 biologically independent mice for each time point). Source data are provided in Source Data file.

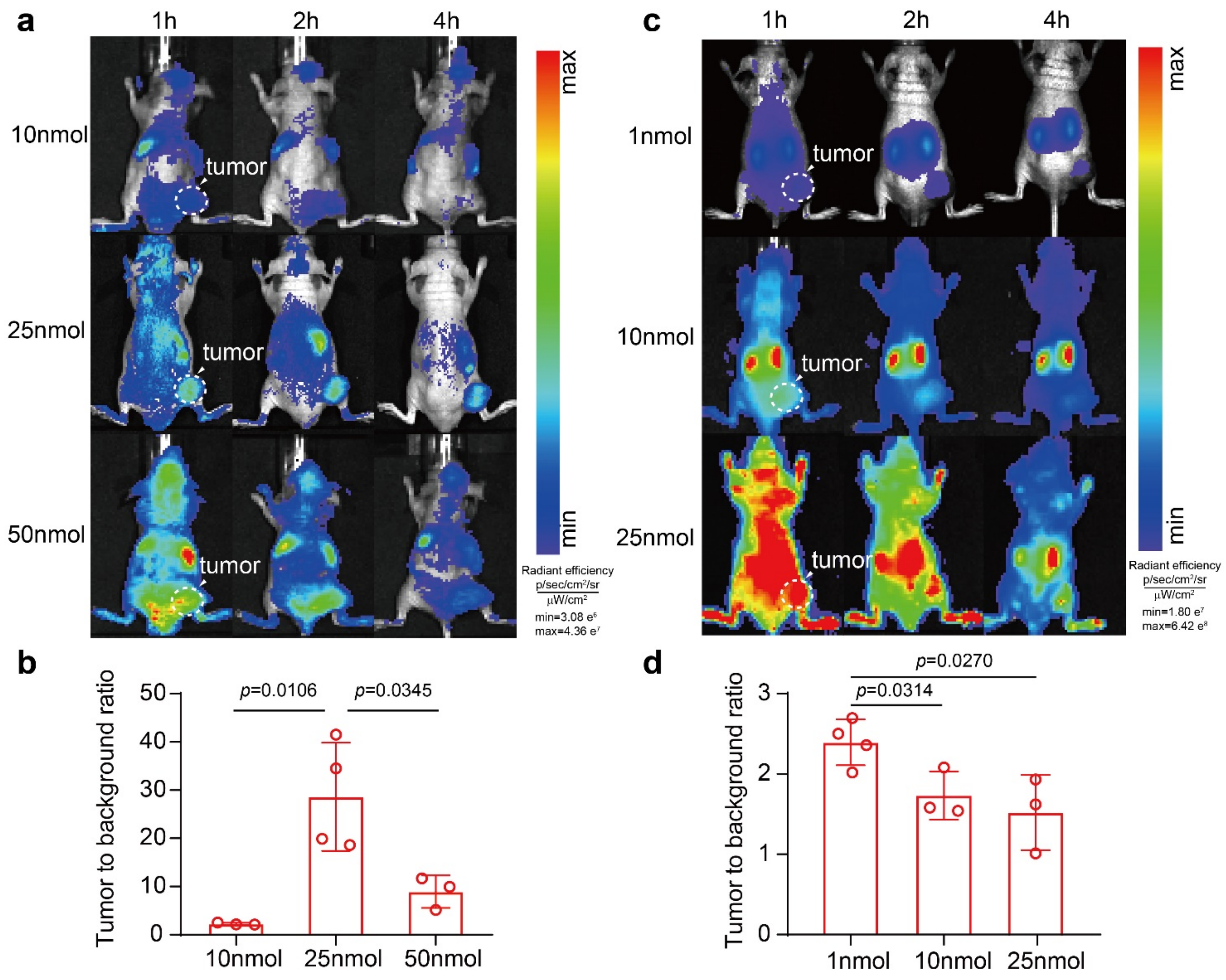

Supplementary Figure 13. In vivo imaging in mouse model of prostate tumor with different doses of probes. (**a**) Fluorescence imaging of 22RV1 tumors after injection with different doses of ODAP-490. Mice were intravenous injected with 10 nmol, 25 nmol or 50 nmol ODAP-490 and images were acquired at 1h, 2h and 4h. The white arrow and dotted circle indicate the location of tumor. (**b**) Quantitative analysis of tumor to background ratio of 22RV1 tumors in panel **a**. Data are shown as mean ± s.d. ($n = 4$ for 25 nmol group, $n = 3$ for 10 nmol and 50 nmol group), two-tailed unpaired Student t-test. (**c**) Fluorescence imaging of 22RV1 tumors after injection with different doses of ODAP-800CW. Mice were intravenous injected with 1 nmol, 10 nmol or 25 nmol ODAP-800CW and images were acquired at 1h, 2h and 4h. The white arrow and dotted circle indicate the location of tumor. (**d**) Quantitative analysis of tumor to background ratio of 22RV1 tumors in panel **c**. Data are shown as mean ± s.d. ($n = 4$ for 1 nmol group, $n = 3$ for 10 nmol and 25 nmol group), two-tailed unpaired Student t-test. Source data are provided in Source Data file.

Full wwPDB X-ray Structure Validation Report (i)

Jan 8, 2021 – 06:03 AM GMT

PDB ID	:	7BFZ
Title	:	X-ray structure of human prostate-specific membrane antigen(PSMA) in complex with a inhibitor Glu-490
Deposited on	:	2021-01-05
Resolution	:	1.73 Å(reported)

This is a Full wwPDB X-ray Structure Validation Report.

This report is produced by the wwPDB biocuration pipeline after annotation of the structure.

We welcome your comments at *validation@mail.wwpdb.org*
A user guide is available at
https://www.wwpdb.org/validation/2017/XrayValidationReportHelp
with specific help available everywhere you see the (i) symbol.

The following versions of software and data (see references (i)) were used in the production of this report:

MolProbity	:	4.02b-467
Mogul	:	1.8.5 (274361), CSD as541be (2020)
Xtriage (Phenix)	:	1.13
EDS	:	2.16
buster-report	:	1.1.7 (2018)
Percentile statistics	:	20191225.v01 (using entries in the PDB archive December 25th 2019)
Refmac	:	5.8.0158
CCP4	:	7.0.044 (Gargrove)
Ideal geometry (proteins)	:	Engh & Huber (2001)
Ideal geometry (DNA, RNA)	:	Parkinson et al. (1996)
Validation Pipeline (wwPDB-VP)	:	2.16

CONFIDENTIAL VALIDATION REPORT

1 Overall quality at a glance (i)

The following experimental techniques were used to determine the structure:
X-RAY DIFFRACTION

The reported resolution of this entry is 1.73 Å.

Percentile scores (ranging between 0-100) for global validation metrics of the entry are shown in the following graphic. The table shows the number of entries on which the scores are based.

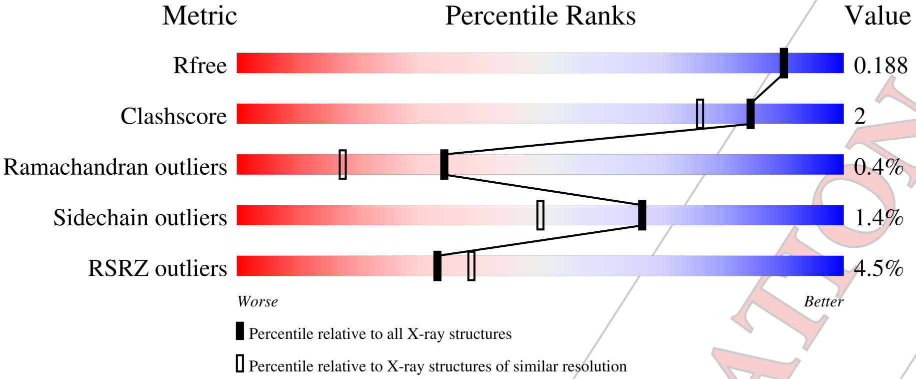

Metric	Whole archive (#Entries)	Similar resolution (#Entries, resolution range(Å))
R_{free}	130704	3764 (1.76-1.72)
Clashscore	141614	3923 (1.76-1.72)
Ramachandran outliers	138981	3878 (1.76-1.72)
Sidechain outliers	138945	3878 (1.76-1.72)
RSRZ outliers	127900	3705 (1.76-1.72)

The table below summarises the geometric issues observed across the polymeric chains and their fit to the electron density. The red, orange, yellow and green segments of the lower bar indicate the fraction of residues that contain outliers for >=3, 2, 1 and 0 types of geometric quality criteria respectively. A grey segment represents the fraction of residues that are not modelled. The numeric value for each fraction is indicated below the corresponding segment, with a dot representing fractions <=5% The upper red bar (where present) indicates the fraction of residues that have poor fit to the electron density. The numeric value is given above the bar.

Mol	Chain	Length	Quality of chain
1	A	707	4% / 91% 7% •
2	B	3	100%
2	F	3	100%
3	C	5	100%
4	D	2	100%

Continued on next page...

CONFIDENTIAL VALIDATION REPORT

Continued from previous page...

Mol	Chain	Length	Quality of chain
5	E	4	100%

The following table lists non-polymeric compounds, carbohydrate monomers and non-standard residues in protein, DNA, RNA chains that are outliers for geometric or electron-density-fit criteria:

Mol	Type	Chain	Res	Chirality	Geometry	Clashes	Electron density
2	BMA	B	3	-	-	-	X
4	NAG	D	2	-	-	-	X
5	BMA	E	3	-	-	-	X
5	FUC	E	4	-	-	-	X

CONFIDENTIAL VALIDATION REPORT

2 Entry composition (i)

There are 14 unique types of molecules in this entry. The entry contains 6622 atoms, of which 0 are hydrogens and 0 are deuteriums.

In the tables below, the ZeroOcc column contains the number of atoms modelled with zero occupancy, the AltConf column contains the number of residues with at least one atom in alternate conformation and the Trace column contains the number of residues modelled with at most 2 atoms.

- Molecule 1 is a protein called Glutamate carboxypeptidase 2.

Mol	Chain	Residues	Atoms					ZeroOcc	AltConf	Trace
1	A	694	Total 5836	C 3753	N 968	O 1093	S 22	0	63	0

- Molecule 2 is an oligosaccharide called beta-D-mannopyranose-(1-4)-2-acetamido-2-deoxy-beta-D-glucopyranose-(1-4)-2-acetamido-2-deoxy-beta-D-glucopyranose.

Mol	Chain	Residues	Atoms				ZeroOcc	AltConf	Trace
2	B	3	Total 39	C 22	N 2	O 15	0	0	0
2	F	3	Total 39	C 22	N 2	O 15	0	0	0

- Molecule 3 is an oligosaccharide called alpha-D-mannopyranose-(1-3)-[alpha-D-mannopyranose-(1-6)]beta-D-mannopyranose-(1-4)-2-acetamido-2-deoxy-beta-D-glucopyranose-(1-4)-2-acetamido-2-deoxy-beta-D-glucopyranose.

Mol	Chain	Residues	Atoms				ZeroOcc	AltConf	Trace
3	C	5	Total 61	C 34	N 2	O 25	0	0	0

- Molecule 4 is an oligosaccharide called 2-acetamido-2-deoxy-beta-D-glucopyranose-(1-4)-2-acetamido-2-deoxy-beta-D-glucopyranose.

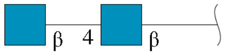

Mol	Chain	Residues	Atoms				ZeroOcc	AltConf	Trace
4	D	2	Total 28	C 16	N 2	O 10	0	0	0

- Molecule 5 is an oligosaccharide called beta-D-mannopyranose-(1-4)-2-acetamido-2-deoxy-b

CONFIDENTIAL VALIDATION REPORT

eta-D-glucopyranose-(1-4)-[alpha-L-fucopyranose-(1-6)]2-acetamido-2-deoxy-beta-D-glucopyranose.

Mol	Chain	Residues	Atoms	ZeroOcc	AltConf	Trace
5	E	4	Total C N O<br>49 28 2 19	0	0	0

- Molecule 6 is ZINC ION (three-letter code: ZN) (formula: Zn).

Mol	Chain	Residues	Atoms	ZeroOcc	AltConf
6	A	2	Total Zn<br>2 2	0	0

- Molecule 7 is CALCIUM ION (three-letter code: CA) (formula: Ca).

Mol	Chain	Residues	Atoms	ZeroOcc	AltConf
7	A	1	Total Ca<br>1 1	0	0

- Molecule 8 is CHLORIDE ION (three-letter code: CL) (formula: Cl).

Mol	Chain	Residues	Atoms	ZeroOcc	AltConf
8	A	1	Total Cl<br>1 1	0	0

- Molecule 9 is 2-acetamido-2-deoxy-beta-D-glucopyranose (three-letter code: NAG) (formula: $C_8H_{15}NO_6$).

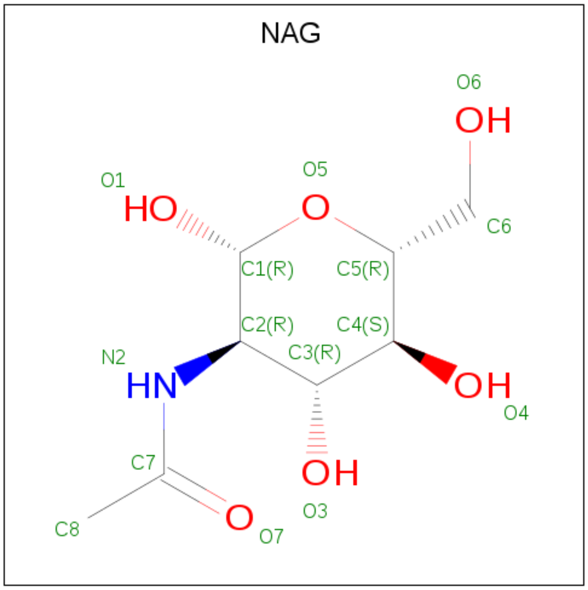

CONFIDENTIAL VALIDATION REPORT

Mol	Chain	Residues	Atoms				ZeroOcc	AltConf
9	A	1	Total 14	C 8	N 1	O 5	0	0
9	A	1	Total 14	C 8	N 1	O 5	0	0

- Molecule 10 is 1,2-ETHANEDIOL (three-letter code: EDO) (formula: $C_2H_6O_2$).

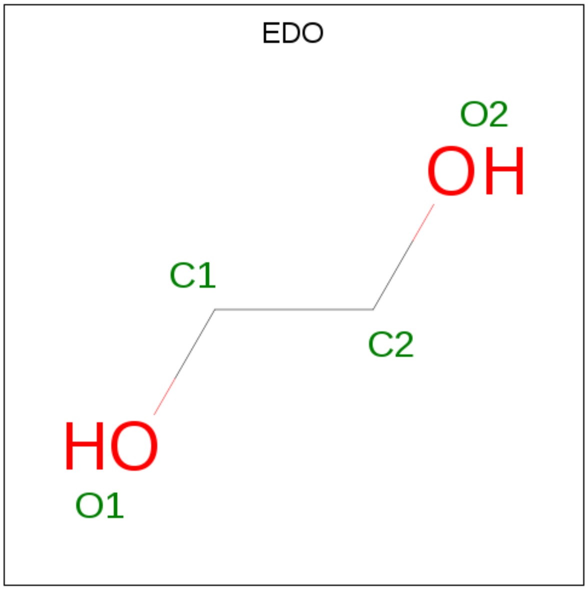

Mol	Chain	Residues	Atoms			ZeroOcc	AltConf
10	A	1	Total 4	C 2	O 2	0	0
10	A	1	Total 4	C 2	O 2	0	0
10	A	1	Total 4	C 2	O 2	0	0

- Molecule 11 is DI(HYDROXYETHYL)ETHER (three-letter code: PEG) (formula: $C_4H_{10}O_3$).

CONFIDENTIAL VALIDATION REPORT

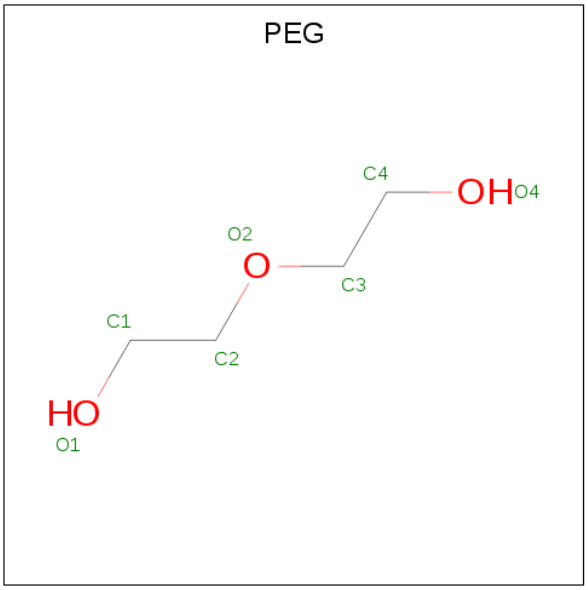

Mol	Chain	Residues	Atoms	ZeroOcc	AltConf
11	A	1	Total C O<br>7 4 3	0	0

- Molecule 12 is SODIUM ION (three-letter code: NA) (formula: Na).

Mol	Chain	Residues	Atoms	ZeroOcc	AltConf
12	A	2	Total Na<br>2 2	0	0

- Molecule 13 is (((S)-1-carboxy-5-((E)-2-cyano-3-(5-(1-(3-methoxy-3-oxopropyl)-1,2,3,4-tetrahydroquinolin-6-yl)thiophen-2-yl)acrylamido)pentyl)carbamoyl)-L-glutamic acid (three-letter code: TKZ) (formula: $C_{33}H_{39}N_5O_{10}S$) (labeled as "Ligand of Interest" by depositor).

CONFIDENTIAL VALIDATION REPORT

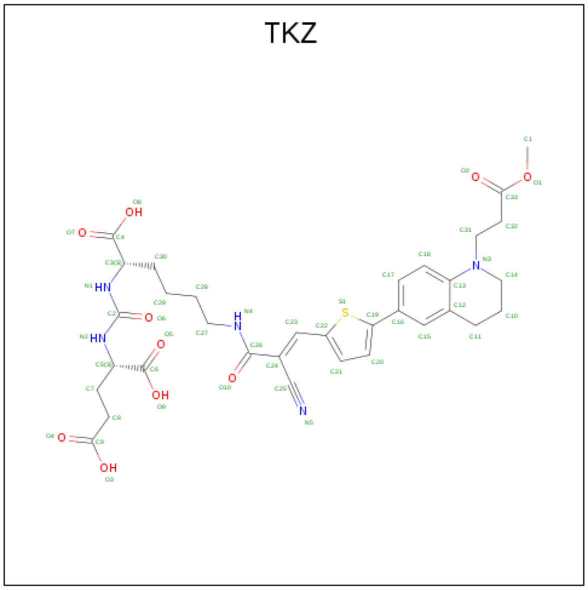

Mol	Chain	Residues	Atoms					ZeroOcc	AltConf
			Total	C	N	O	S		
13	A	1	49	33	5	10	1	0	0

- Molecule 14 is water.

Mol	Chain	Residues	Atoms		ZeroOcc	AltConf
			Total	O		
14	A	467	468	468	0	1

CONFIDENTIAL VALIDATION REPORT

3 Residue-property plots ⓘ

These plots are drawn for all protein, RNA, DNA and oligosaccharide chains in the entry. The first graphic for a chain summarises the proportions of the various outlier classes displayed in the second graphic. The second graphic shows the sequence view annotated by issues in geometry and electron density. Residues are color-coded according to the number of geometric quality criteria for which they contain at least one outlier: green = 0, yellow = 1, orange = 2 and red = 3 or more. A red dot above a residue indicates a poor fit to the electron density (RSRZ > 2). Stretches of 2 or more consecutive residues without any outlier are shown as a green connector. Residues present in the sample, but not in the model, are shown in grey.

• Molecule 1: Glutamate carboxypeptidase 2

Chain A:

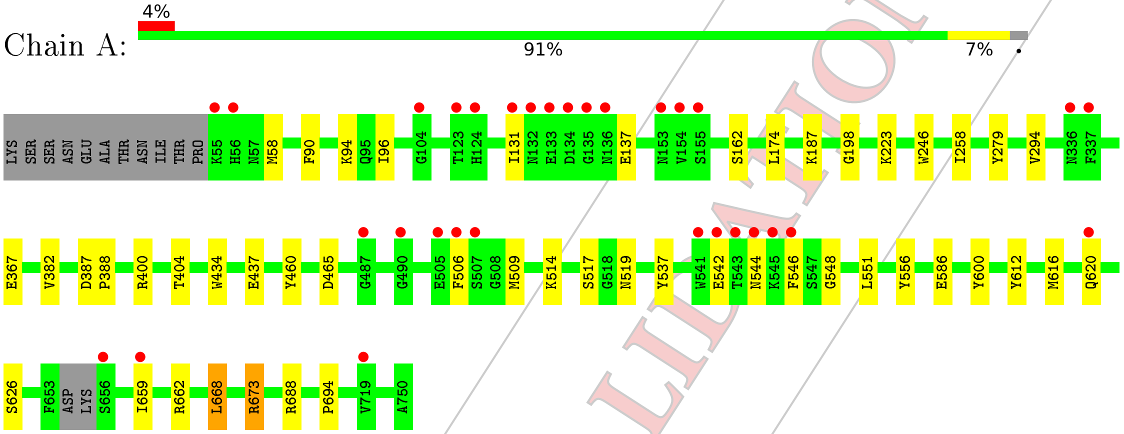

• Molecule 2: beta-D-mannopyranose-(1-4)-2-acetamido-2-deoxy-beta-D-glucopyranose-(1-4)-2-acetamido-2-deoxy-beta-D-glucopyranose

Chain B:

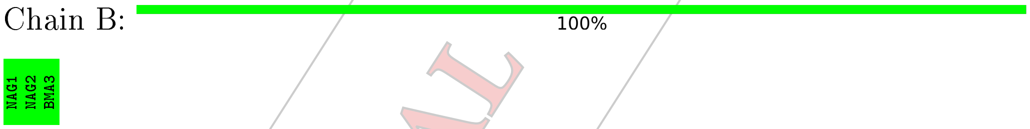

• Molecule 2: beta-D-mannopyranose-(1-4)-2-acetamido-2-deoxy-beta-D-glucopyranose-(1-4)-2-acetamido-2-deoxy-beta-D-glucopyranose

Chain F:

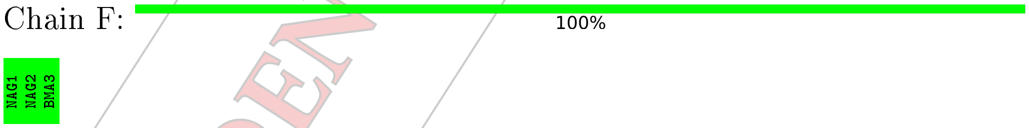

• Molecule 3: alpha-D-mannopyranose-(1-3)-[alpha-D-mannopyranose-(1-6)]beta-D-mannopyranose-(1-4)-2-acetamido-2-deoxy-beta-D-glucopyranose-(1-4)-2-acetamido-2-deoxy-beta-D-glucopyranose

Chain C:

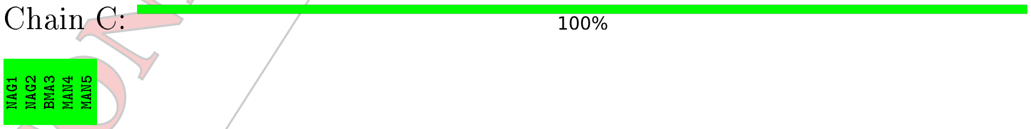

CONFIDENTIAL VALIDATION REPORT

• Molecule 4: 2-acetamido-2-deoxy-beta-D-glucopyranose-(1-4)-2-acetamido-2-deoxy-beta-D-glucopyranose

Chain D: 100%

NAG1 NAG2

• Molecule 5: beta-D-mannopyranose-(1-4)-2-acetamido-2-deoxy-beta-D-glucopyranose-(1-4)-[alpha-L-fucopyranose-(1-6)]2-acetamido-2-deoxy-beta-D-glucopyranose

Chain E: 100%

NAG1 NAG2 BMA3 FUC4

CONFIDENTIAL VALIDATION REPORT

4 Data and refinement statistics (i)

Property	Value	Source
Space group	I 2 2 2	Depositor
Cell constants<br>a, b, c, α, β, γ	101.54Å 130.10Å 158.91Å<br>90.00° 90.00° 90.00°	Depositor
Resolution (Å)	46.96 – 1.73<br>46.96 – 1.73	Depositor<br>EDS
% Data completeness<br>(in resolution range)	99.5 (46.96-1.73)<br>99.5 (46.96-1.73)	Depositor<br>EDS
R_{merge}	(Not available)	Depositor
R_{sym}	(Not available)	Depositor
$< I/\sigma(I) >$ [1]	1.75 (at 1.73Å)	Xtriage
Refinement program	REFMAC 5.8.0135	Depositor
R, R_{free}	0.163 , 0.183<br>0.171 , 0.188	Depositor<br>DCC
R_{free} test set	2101 reflections (1.93%)	wwPDB-VP
Wilson B-factor (Å^2)	23.9	Xtriage
Anisotropy	0.576	Xtriage
Bulk solvent k_{sol}(e/Å^3), B_{sol}(Å^2)	0.36 , 47.2	EDS
L-test for twinning[2]	$< \|L\| > = 0.50$, $< L^2 > = 0.34$	Xtriage
Estimated twinning fraction	No twinning to report.	Xtriage
F_o,F_c correlation	0.97	EDS
Total number of atoms	6622	wwPDB-VP
Average B, all atoms (Å^2)	36.0	wwPDB-VP

Xtriage's analysis on translational NCS is as follows: *The largest off-origin peak in the Patterson function is 4.19% of the height of the origin peak. No significant pseudotranslation is detected.*

[1] Intensities estimated from amplitudes.

[2] Theoretical values of $< |L| >$, $< L^2 >$ for acentric reflections are 0.5, 0.333 respectively for untwinned datasets, and 0.375, 0.2 for perfectly twinned datasets.

CONFIDENTIAL VALIDATION REPORT

5 Model quality (i)

5.1 Standard geometry (i)

Bond lengths and bond angles in the following residue types are not validated in this section: ZN, BMA, NAG, CL, NA, CA, EDO, FUC, PEG, TKZ, MAN

The Z score for a bond length (or angle) is the number of standard deviations the observed value is removed from the expected value. A bond length (or angle) with $|Z| > 5$ is considered an outlier worth inspection. RMSZ is the root-mean-square of all Z scores of the bond lengths (or angles).

Mol	Chain	Bond lengths		Bond angles	
		RMSZ	$\#\|Z\| > 5$	RMSZ	$\#\|Z\| > 5$
1	A	0.91	2/6177 (0.0%)	0.85	4/8361 (0.0%)

Chiral center outliers are detected by calculating the chiral volume of a chiral center and verifying if the center is modelled as a planar moiety or with the opposite hand.A planarity outlier is detected by checking planarity of atoms in a peptide group, atoms in a mainchain group or atoms of a sidechain that are expected to be planar.

Mol	Chain	#Chirality outliers	#Planarity outliers
1	A	0	1

All (2) bond length outliers are listed below:

Mol	Chain	Res	Type	Atoms	Z	Observed(Å)	Ideal(Å)
1	A	434	TRP	CE3-CZ3	5.43	1.47	1.38
1	A	460	TYR	CG-CD1	5.11	1.45	1.39

All (4) bond angle outliers are listed below:

Mol	Chain	Res	Type	Atoms	Z	Observed(^{o})	Ideal(^{o})
1	A	465	ASP	CB-CG-OD1	6.33	123.99	118.30
1	A	673	ARG	NE-CZ-NH1	5.33	122.97	120.30
1	A	668[A]	LEU	CB-CG-CD1	5.16	119.77	111.00
1	A	668[B]	LEU	CB-CG-CD1	5.16	119.77	111.00

There are no chirality outliers.

All (1) planarity outliers are listed below:

Mol	Chain	Res	Type	Group
1	A	546	PHE	Peptide

CONFIDENTIAL VALIDATION REPORT

5.2 Too-close contacts (i)

In the following table, the Non-H and H(model) columns list the number of non-hydrogen atoms and hydrogen atoms in the chain respectively. The H(added) column lists the number of hydrogen atoms added and optimized by MolProbity. The Clashes column lists the number of clashes within the asymmetric unit, whereas Symm-Clashes lists symmetry-related clashes.

Mol	Chain	Non-H	H(model)	H(added)	Clashes	Symm-Clashes
1	A	5836	0	5757	28	0
2	B	39	0	34	0	0
2	F	39	0	34	0	0
3	C	61	0	52	0	0
4	D	28	0	25	0	0
5	E	49	0	43	0	0
6	A	2	0	0	0	0
7	A	1	0	0	0	0
8	A	1	0	0	0	0
9	A	28	0	26	1	0
10	A	12	0	18	0	0
11	A	7	0	10	0	0
12	A	2	0	0	0	0
13	A	49	0	0	1	0
14	A	468	0	0	7	0
All	All	6622	0	5999	28	0

The all-atom clashscore is defined as the number of clashes found per 1000 atoms (including hydrogen atoms). The all-atom clashscore for this structure is 2.

All (28) close contacts within the same asymmetric unit are listed below, sorted by their clash magnitude.

Atom-1	Atom-2	Interatomic distance (Å)	Clash overlap (Å)
1:A:437[B]:GLU:OE1	14:A:902:HOH:O	1.76	1.01
1:A:620[B]:GLN:NE2	14:A:901:HOH:O	1.58	0.82
1:A:620[A]:GLN:NE2	14:A:901:HOH:O	2.21	0.72
1:A:58:MET:CE	1:A:586:GLU:HG2	2.32	0.59
1:A:400:ARG:O	1:A:404[B]:THR:HG23	2.02	0.58
1:A:162:SER:HB3	1:A:258[B]:ILE:HD13	1.86	0.58
1:A:612:TYR:CZ	1:A:616:MET:HG3	2.39	0.57
1:A:688[B]:ARG:NH1	14:A:903:HOH:O	1.86	0.55
1:A:131[B]:ILE:HG23	1:A:131[B]:ILE:O	2.07	0.55
1:A:58:MET:HE1	1:A:586:GLU:HG2	1.92	0.50
1:A:90:PHE:CE2	1:A:94:LYS:HE2	2.46	0.50
1:A:367:GLU:OE1	1:A:662[A]:ARG:NH1	2.42	0.48

Continued on next page...

CONFIDENTIAL VALIDATION REPORT

Continued from previous page...

Atom-1	Atom-2	Interatomic distance (Å)	Clash overlap (Å)
1:A:96[B]:ILE:HD13	14:A:942:HOH:O	2.12	0.48
1:A:131[B]:ILE:CG2	1:A:131[B]:ILE:O	2.63	0.47
1:A:626[B]:SER:HA	14:A:1082:HOH:O	2.15	0.46
1:A:626[A]:SER:HA	14:A:1082:HOH:O	2.16	0.45
1:A:258[A]:ILE:HD13	1:A:294:VAL:HB	1.99	0.45
1:A:246:TRP:CD1	9:A:806:NAG:H83	2.52	0.44
1:A:131[A]:ILE:HG22	1:A:137:GLU:HG2	1.99	0.44
1:A:659[B]:ILE:HA	1:A:659[B]:ILE:HD13	1.67	0.44
1:A:506:PHE:HB2	1:A:509[A]:MET:HG3	2.00	0.43
1:A:198:GLY:O	1:A:223:LYS:HE2	2.18	0.43
1:A:551:LEU:HD22	1:A:556:TYR:HB2	2.00	0.42
1:A:58:MET:HE2	1:A:586:GLU:HG2	1.99	0.42
1:A:514[A]:LYS:HG3	13:A:813:TKZ:C1	2.49	0.42
1:A:387:ASP:HA	1:A:388:PRO:HA	1.89	0.41
1:A:517[A]:SER:HB2	1:A:694:PRO:HG3	2.02	0.41

There are no symmetry-related clashes.

5.3 Torsion angles (i)

5.3.1 Protein backbone (i)

In the following table, the Percentiles column shows the percent Ramachandran outliers of the chain as a percentile score with respect to all X-ray entries followed by that with respect to entries of similar resolution.

The Analysed column shows the number of residues for which the backbone conformation was analysed, and the total number of residues.

Mol	Chain	Analysed	Favoured	Allowed	Outliers	Percentiles	
1	A	754/707 (107%)	735 (98%)	16 (2%)	3 (0%)	34	17

All (3) Ramachandran outliers are listed below:

Mol	Chain	Res	Type
1	A	544	ASN
1	A	382	VAL
1	A	548	GLY

CONFIDENTIAL VALIDATION REPORT

5.3.2 Protein sidechains (i)

In the following table, the Percentiles column shows the percent sidechain outliers of the chain as a percentile score with respect to all X-ray entries followed by that with respect to entries of similar resolution.

The Analysed column shows the number of residues for which the sidechain conformation was analysed, and the total number of residues.

Mol	Chain	Analysed	Rotameric	Outliers	Percentiles	
1	A	656/603 (109%)	647 (99%)	9 (1%)	67	50

All (9) residues with a non-rotameric sidechain are listed below:

Mol	Chain	Res	Type
1	A	174	LEU
1	A	187	LYS
1	A	519	ASN
1	A	537	TYR
1	A	542	GLU
1	A	600	TYR
1	A	668[A]	LEU
1	A	668[B]	LEU
1	A	673	ARG

Sometimes sidechains can be flipped to improve hydrogen bonding and reduce clashes. All (1) such sidechains are listed below:

Mol	Chain	Res	Type
1	A	136	ASN

5.3.3 RNA (i)

There are no RNA molecules in this entry.

5.4 Non-standard residues in protein, DNA, RNA chains (i)

There are no non-standard protein/DNA/RNA residues in this entry.

5.5 Carbohydrates (i)

17 monosaccharides are modelled in this entry.

CONFIDENTIAL VALIDATION REPORT

There are no bond length outliers.

There are no bond angle outliers.

There are no chirality outliers.

There are no torsion outliers.

There are no ring outliers.

No monomer is involved in short contacts.

The following is a two-dimensional graphical depiction of Mogul quality analysis of bond lengths, bond angles, torsion angles, and ring geometry for oligosaccharide.

Oligosaccharide Chain B

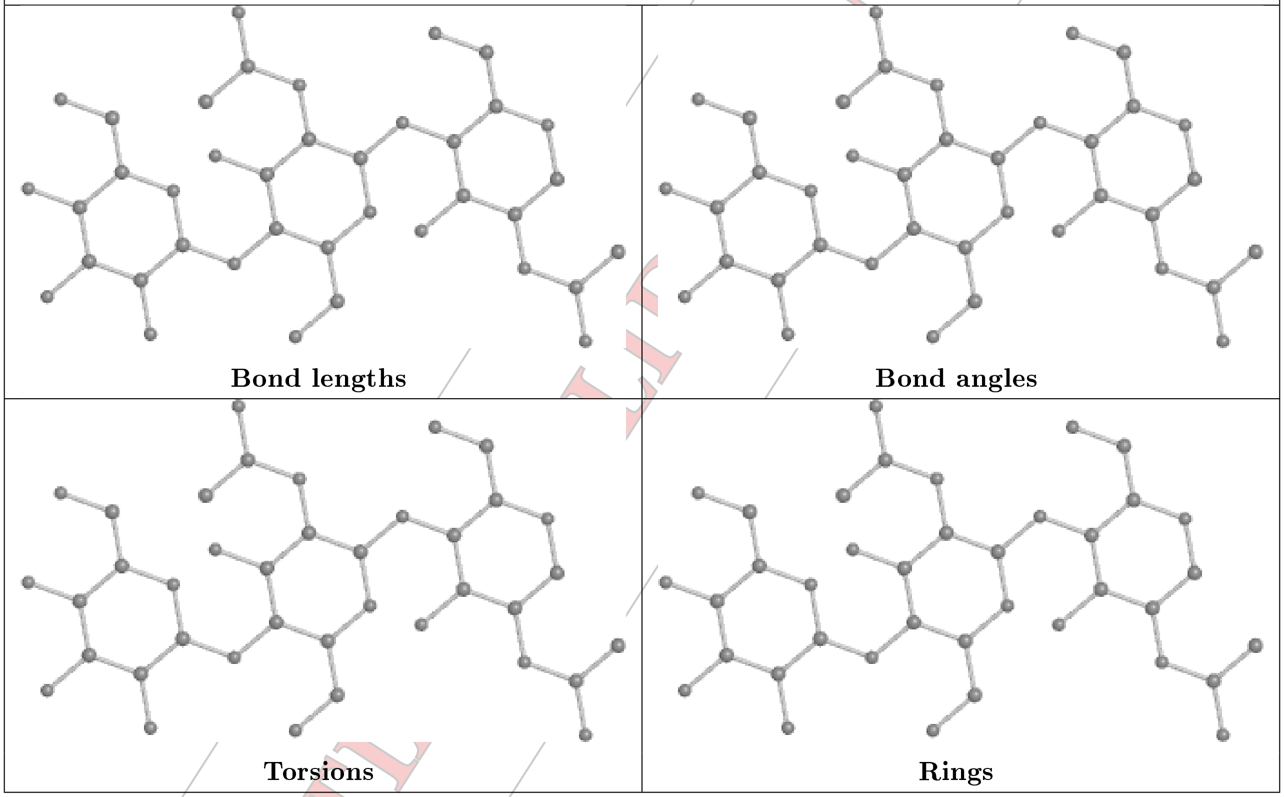

CONFIDENTIAL REPORT

Oligosaccharide Chain F

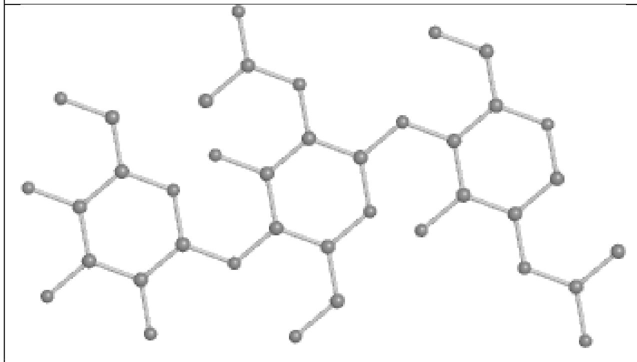

Bond lengths

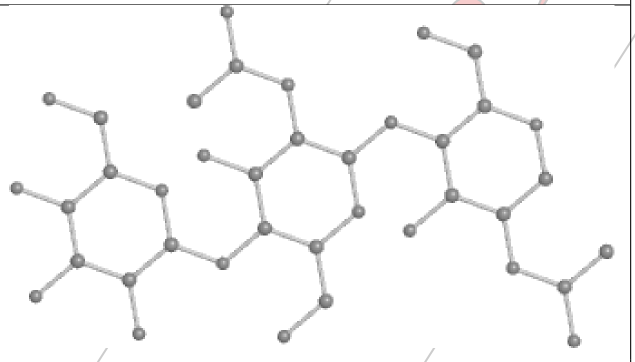

Bond angles

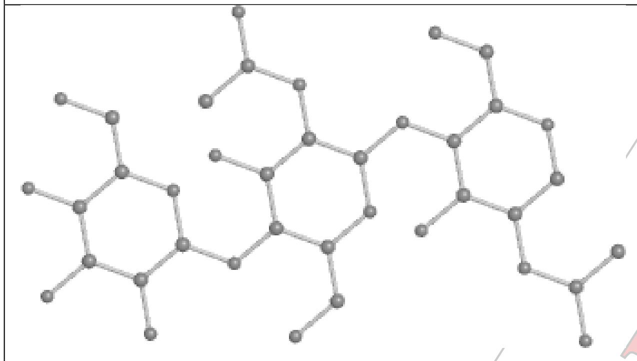

Torsions

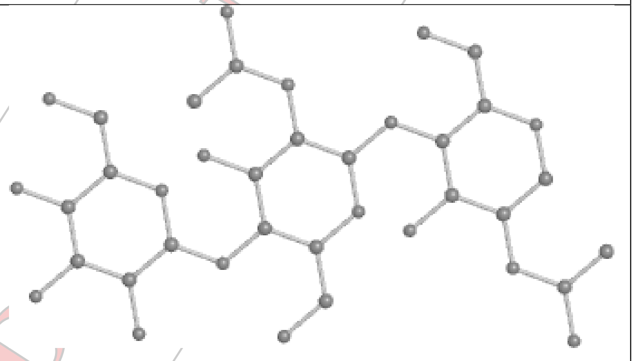

Rings

Oligosaccharide Chain C

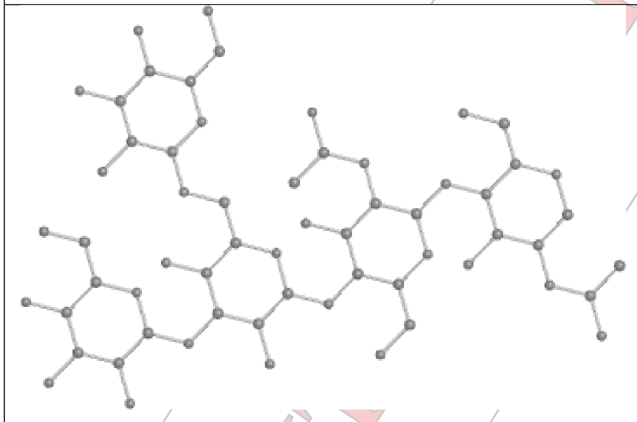

Bond lengths

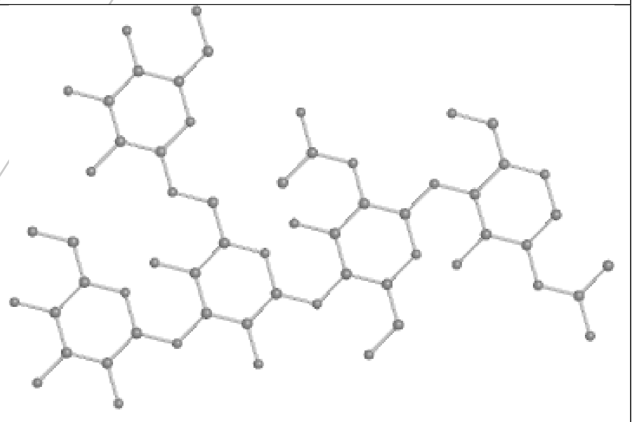

Bond angles

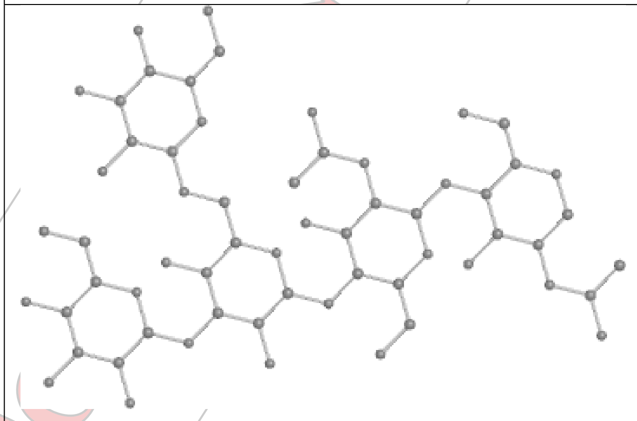

Torsions

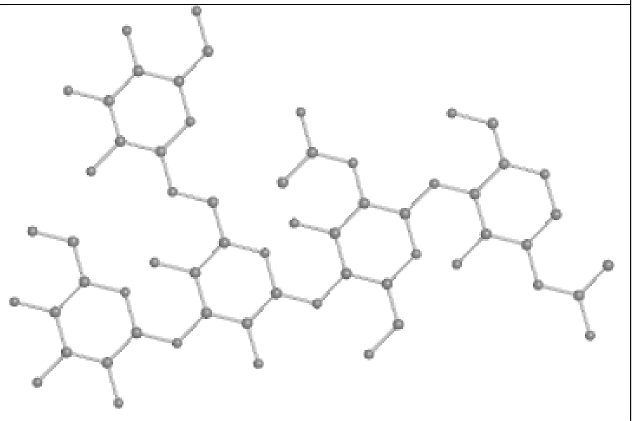

Rings

Oligosaccharide Chain D

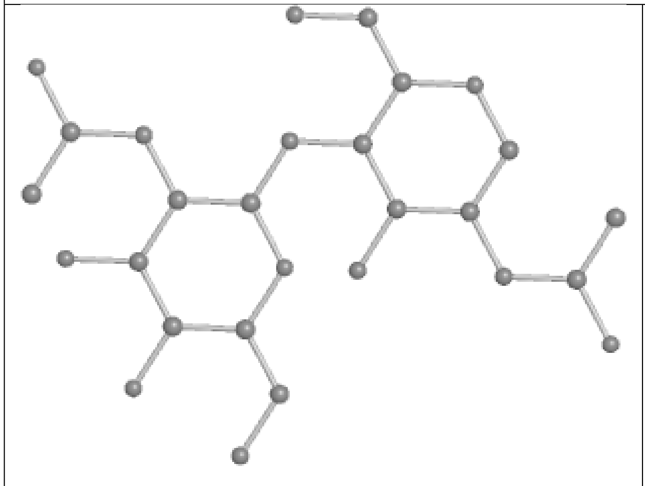

Bond lengths

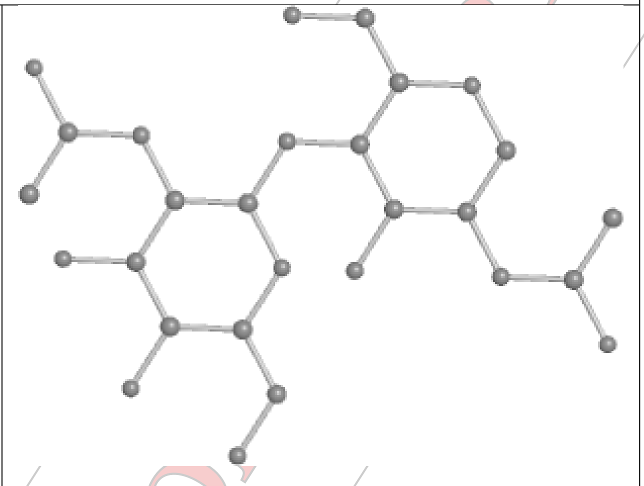

Bond angles

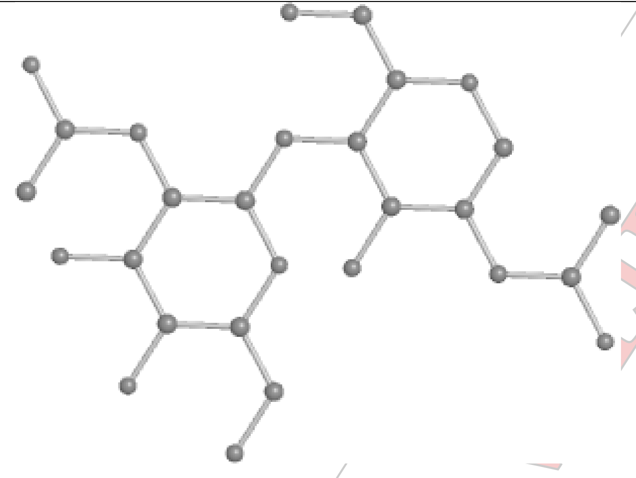

Torsions

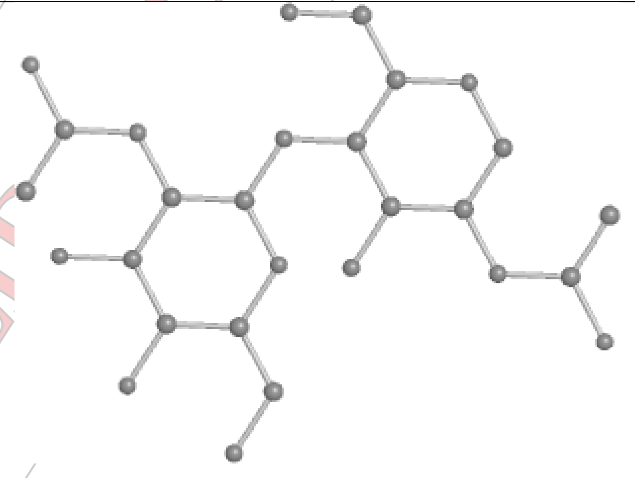

Rings

CONFIDENTIAL

Oligosaccharide Chain E

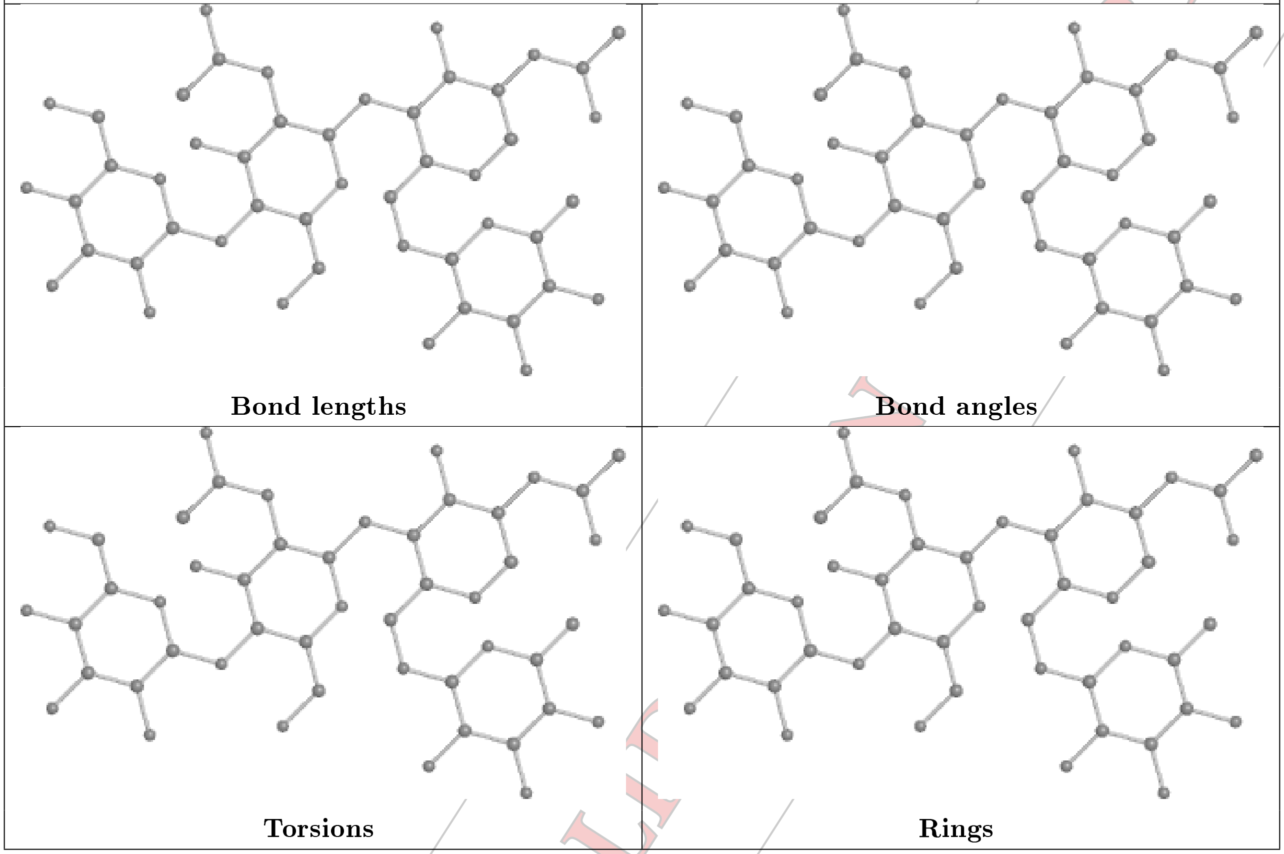

5.6 Ligand geometry ⓘ

Of 13 ligands modelled in this entry, 6 are monoatomic - leaving 7 for Mogul analysis.

There are no bond length outliers.

There are no bond angle outliers.

There are no chirality outliers.

There are no torsion outliers.

There are no ring outliers.

No monomer is involved in short contacts.

The following is a two-dimensional graphical depiction of Mogul quality analysis of bond lengths, bond angles, torsion angles, and ring geometry for all instances of the Ligand of Interest. In addition, ligands with molecular weight > 250 and outliers as shown on the validation Tables will also be included. For torsion angles, if less then 5% of the Mogul distribution of torsion angles is within 10 degrees of the torsion angle in question, then that torsion angle is considered an outlier. Any bond that is central to one or more torsion angles identified as an outlier by Mogul will be highlighted in the graph. For rings, the root-mean-square deviation (RMSD) between the ring in question and similar rings identified by Mogul is calculated over all ring torsion angles. If the average RMSD is greater than 60 degrees and the minimal RMSD between the ring in question and

CONFIDENTIAL VALIDATION REPORT

any Mogul-identified rings is also greater than 60 degrees, then that ring is considered an outlier. The outliers are highlighted in purple. The color gray indicates Mogul did not find sufficient equivalents in the CSD to analyse the geometry.

**Ligand TKZ A 813**	
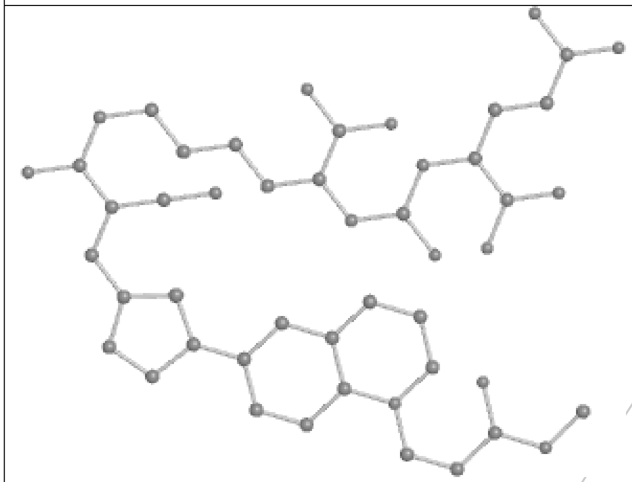 **Bond lengths**	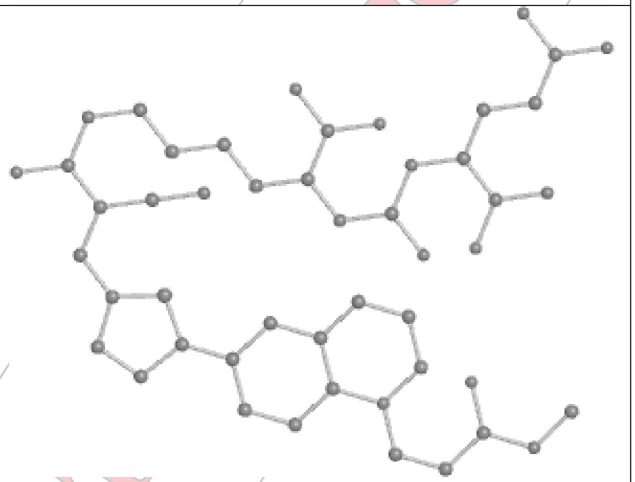 **Bond angles**
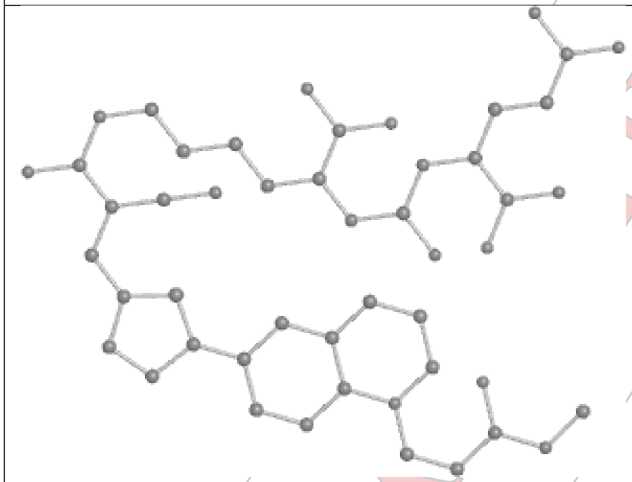 **Torsions**	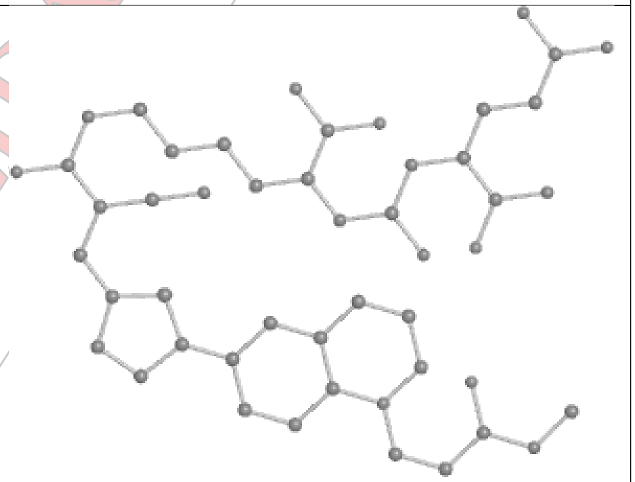 **Rings**

5.7 Other polymers (i)

There are no such residues in this entry.

5.8 Polymer linkage issues (i)

There are no chain breaks in this entry.

CONFIDENTIAL REPORT

6 Fit of model and data (i)

6.1 Protein, DNA and RNA chains (i)

In the following table, the column labelled '#RSRZ> 2' contains the number (and percentage) of RSRZ outliers, followed by percent RSRZ outliers for the chain as percentile scores relative to all X-ray entries and entries of similar resolution. The OWAB column contains the minimum, median, 95th percentile and maximum values of the occupancy-weighted average B-factor per residue. The column labelled 'Q< 0.9' lists the number of (and percentage) of residues with an average occupancy less than 0.9.

Mol	Chain	Analysed	<RSRZ>	#RSRZ>2			OWAB(Å^2)	Q<0.9
1	A	694/707 (98%)	0.22	31 (4%)	33	38	22, 32, 53, 97	0

All (31) RSRZ outliers are listed below:

Mol	Chain	Res	Type	RSRZ
1	A	546	PHE	10.3
1	A	543	THR	4.7
1	A	155	SER	4.5
1	A	507	SER	4.3
1	A	55	LYS	4.3
1	A	656[A]	SER	4.0
1	A	153	ASN	3.8
1	A	154	VAL	3.5
1	A	542	GLU	3.5
1	A	506	PHE	3.4
1	A	487	GLY	3.3
1	A	135	GLY	3.2
1	A	136	ASN	3.1
1	A	719	VAL	3.1
1	A	131[A]	ILE	2.9
1	A	337	PHE	2.8
1	A	134	ASP	2.8
1	A	544	ASN	2.8
1	A	541	TRP	2.7
1	A	124	HIS	2.7
1	A	56	HIS	2.6
1	A	620[A]	GLN	2.5
1	A	123	THR	2.4
1	A	659[A]	ILE	2.3
1	A	490	GLY	2.2
1	A	104	GLY	2.1
1	A	545	LYS	2.1

Continued on next page...

CONFIDENTIAL VALIDATION REPORT

Continued from previous page...

Mol	Chain	Res	Type	RSRZ
1	A	133	GLU	2.1
1	A	505	GLU	2.1
1	A	336	ASN	2.1
1	A	132	ASN	2.0

6.2 Non-standard residues in protein, DNA, RNA chains (i)

There are no non-standard protein/DNA/RNA residues in this entry.

6.3 Carbohydrates (i)

In the following table, the Atoms column lists the number of modelled atoms in the group and the number defined in the chemical component dictionary. The B-factors column lists the minimum, median, 95th percentile and maximum values of B factors of atoms in the group. The column labelled 'Q< 0.9' lists the number of atoms with occupancy less than 0.9.

Mol	Type	Chain	Res	Atoms	RSCC	RSR	B-factors(Å^2)	Q<0.9
2	BMA	B	3	11/12	0.52	0.54	93,100,105,105	0
5	BMA	E	3	11/12	0.60	0.51	109,116,121,124	0
2	NAG	B	2	14/15	0.68	0.32	46,61,72,83	0
2	BMA	F	3	11/12	0.70	0.27	69,74,82,89	0
5	FUC	E	4	10/11	0.72	0.44	74,81,84,86	0
4	NAG	D	1	14/15	0.73	0.25	57,65,82,89	0
4	NAG	D	2	14/15	0.74	0.45	88,92,96,104	0
5	NAG	E	1	14/15	0.82	0.20	46,51,57,62	0
3	MAN	C	5	11/12	0.85	0.25	67,74,84,91	0
5	NAG	E	2	14/15	0.85	0.33	59,69,81,98	0
3	NAG	C	1	14/15	0.89	0.11	27,35,44,57	0
3	BMA	C	3	11/12	0.91	0.10	45,47,53,54	0
3	NAG	C	2	14/15	0.91	0.17	44,47,54,64	0
2	NAG	F	2	14/15	0.93	0.13	42,51,59,64	0
3	MAN	C	4	11/12	0.93	0.10	49,53,56,56	0
2	NAG	F	1	14/15	0.93	0.10	34,37,40,48	0
2	NAG	B	1	14/15	0.94	0.15	38,45,51,56	0

The following is a graphical depiction of the model fit to experimental electron density for oligosaccharide. Each fit is shown from different orientation to approximate a three-dimensional view.

CONFIDENTIAL VALIDATION REPORT

Electron density around Chain B:

$2mF_o$-DF_c (at 0.7 rmsd) in gray
mF_o-DF_c (at 3 rmsd) in purple (negative)
and green (positive)

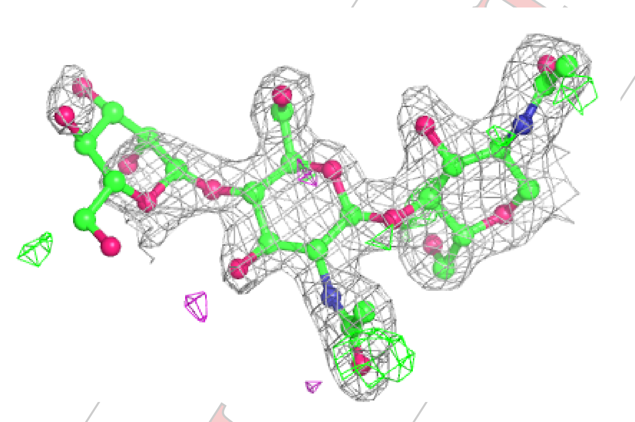

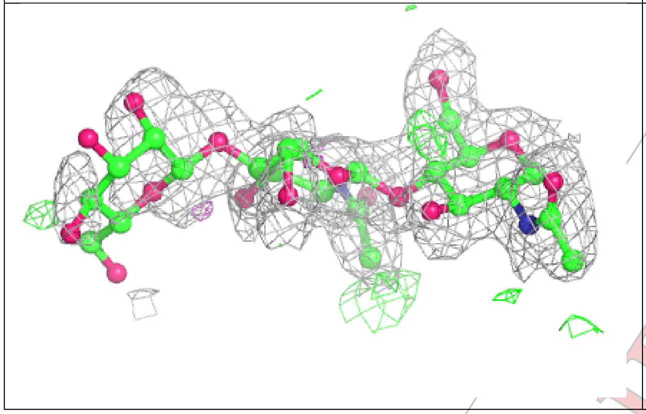

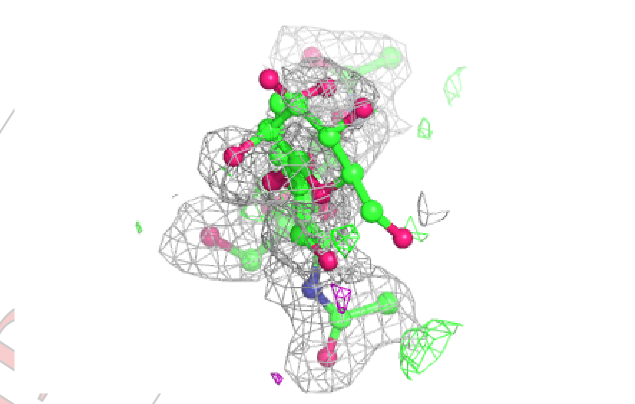

Electron density around Chain F:

$2mF_o$-DF_c (at 0.7 rmsd) in gray
mF_o-DF_c (at 3 rmsd) in purple (negative)
and green (positive)

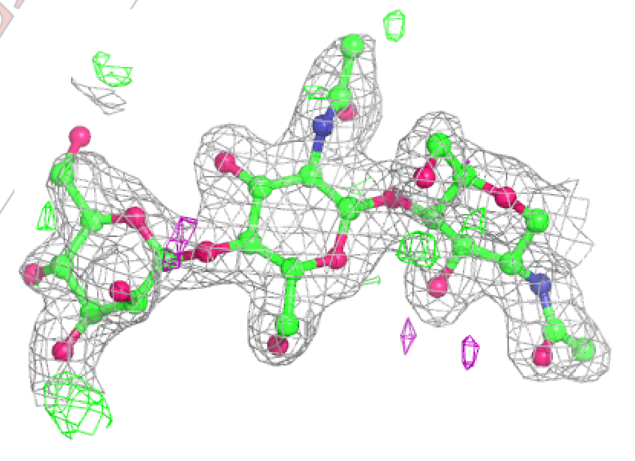

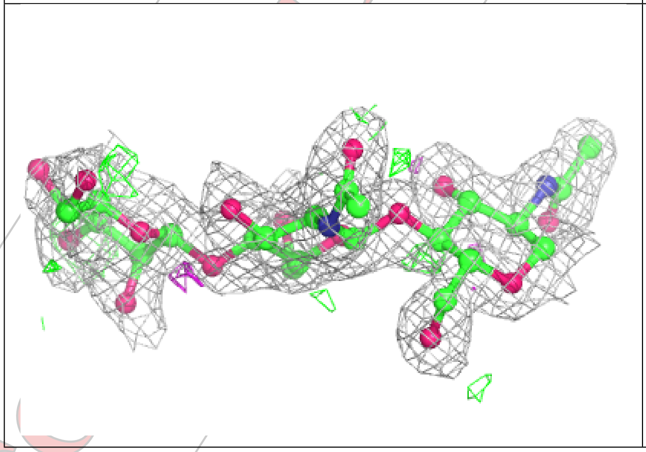

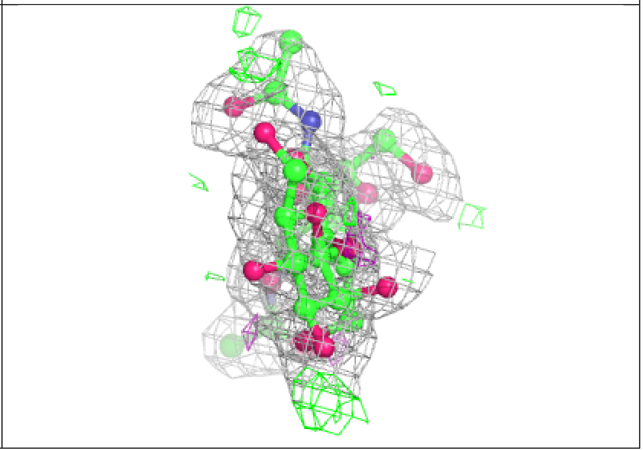

Electron density around Chain C:

2mF$_o$-DF$_c$ (at 0.7 rmsd) in gray
mF$_o$-DF$_c$ (at 3 rmsd) in purple (negative)
and green (positive)

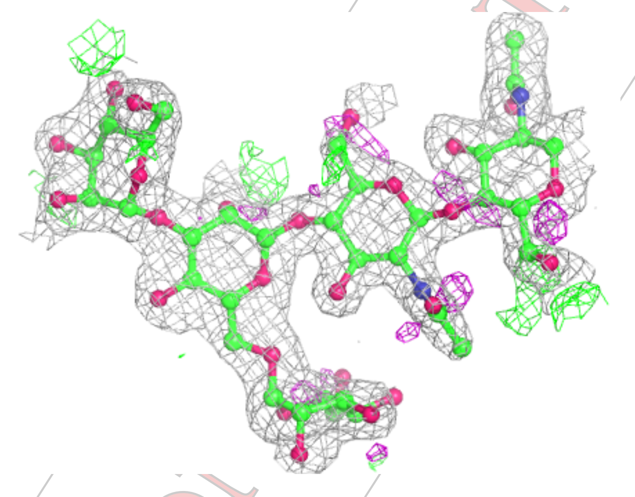

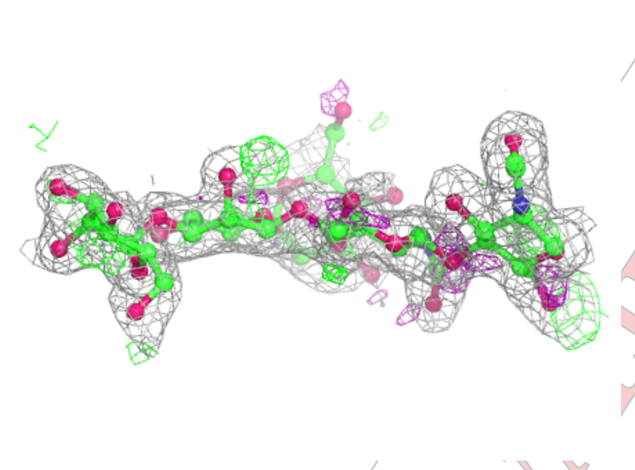

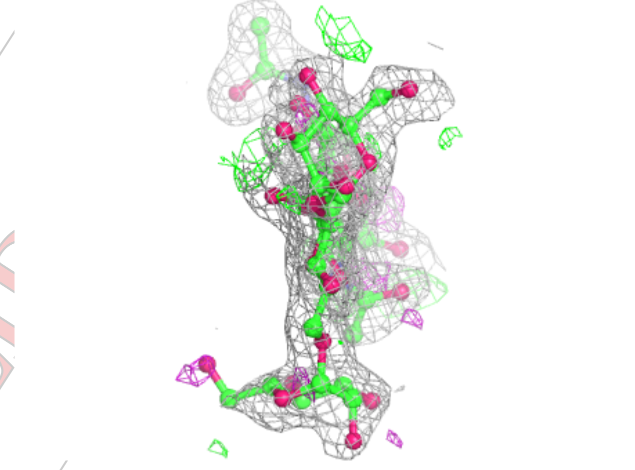

CONFIDENTIAL VALIDATION REPORT

Electron density around Chain D:

$2mF_o$-DF_c (at 0.7 rmsd) in gray
mF_o-DF_c (at 3 rmsd) in purple (negative)
and green (positive)

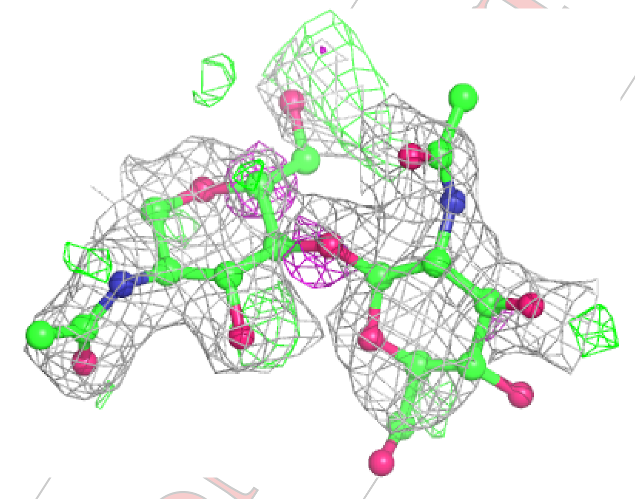

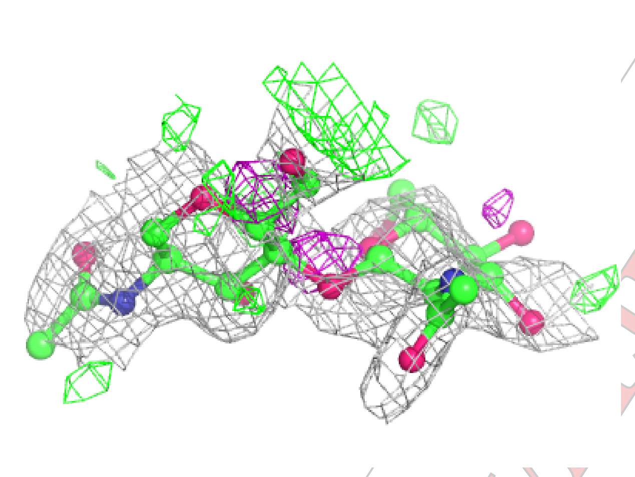

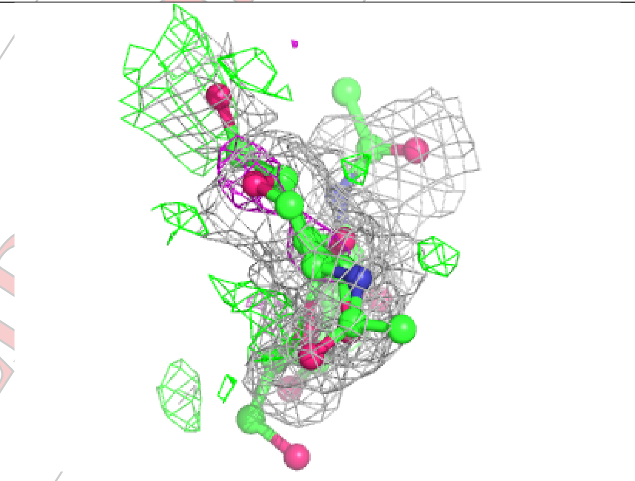

CONFIDENTIAL

Electron density around Chain E:

$2mF_o$-DF_c (at 0.7 rmsd) in gray
mF_o-DF_c (at 3 rmsd) in purple (negative)
and green (positive)

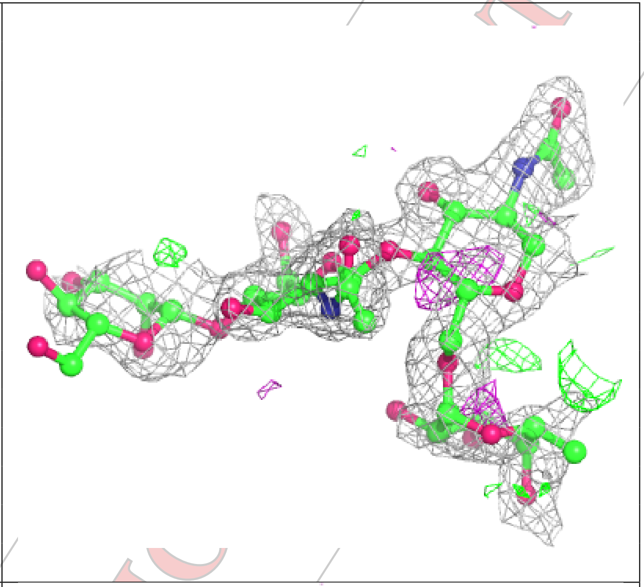

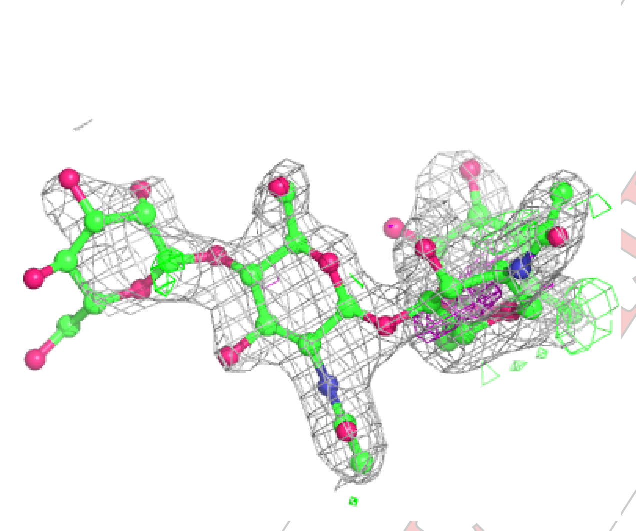

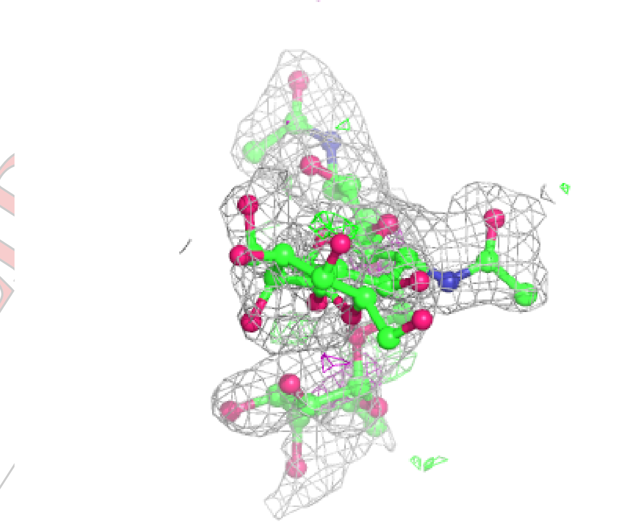

6.4 Ligands (i)

In the following table, the Atoms column lists the number of modelled atoms in the group and the number defined in the chemical component dictionary. The B-factors column lists the minimum, median, 95th percentile and maximum values of B factors of atoms in the group. The column labelled ‘Q< 0.9’ lists the number of atoms with occupancy less than 0.9.

Mol	Type	Chain	Res	Atoms	RSCC	RSR	B-factors($Å^2$)	Q<0.9
12	NA	A	811	1/1	0.73	0.22	55,55,55,55	0
9	NAG	A	805	14/15	0.81	0.28	67,77,84,84	0
10	EDO	A	807	4/4	0.85	0.32	49,57,60,62	0
9	NAG	A	806	14/15	0.88	0.12	37,52,57,60	0
12	NA	A	812	1/1	0.89	0.36	57,57,57,57	0
10	EDO	A	808	4/4	0.89	0.18	50,57,59,62	0
11	PEG	A	809	7/7	0.90	0.11	61,62,68,69	0

Continued on next page...

CONFIDENTIAL

Continued from previous page...

Mol	Type	Chain	Res	Atoms	RSCC	RSR	B-factors(Å^2)	Q<0.9
10	EDO	A	810	4/4	0.91	0.24	44,55,58,64	0
13	TKZ	A	813	49/49	0.92	0.16	23,45,69,77	0
8	CL	A	804	1/1	0.99	0.17	26,26,26,26	0
6	ZN	A	802	1/1	1.00	0.10	25,25,25,25	0
7	CA	A	803	1/1	1.00	0.09	24,24,24,24	0
6	ZN	A	801	1/1	1.00	0.08	25,25,25,25	0

The following is a graphical depiction of the model fit to experimental electron density of all instances of the Ligand of Interest. In addition, ligands with molecular weight > 250 and outliers as shown on the geometry validation Tables will also be included. Each fit is shown from different orientation to approximate a three-dimensional view.

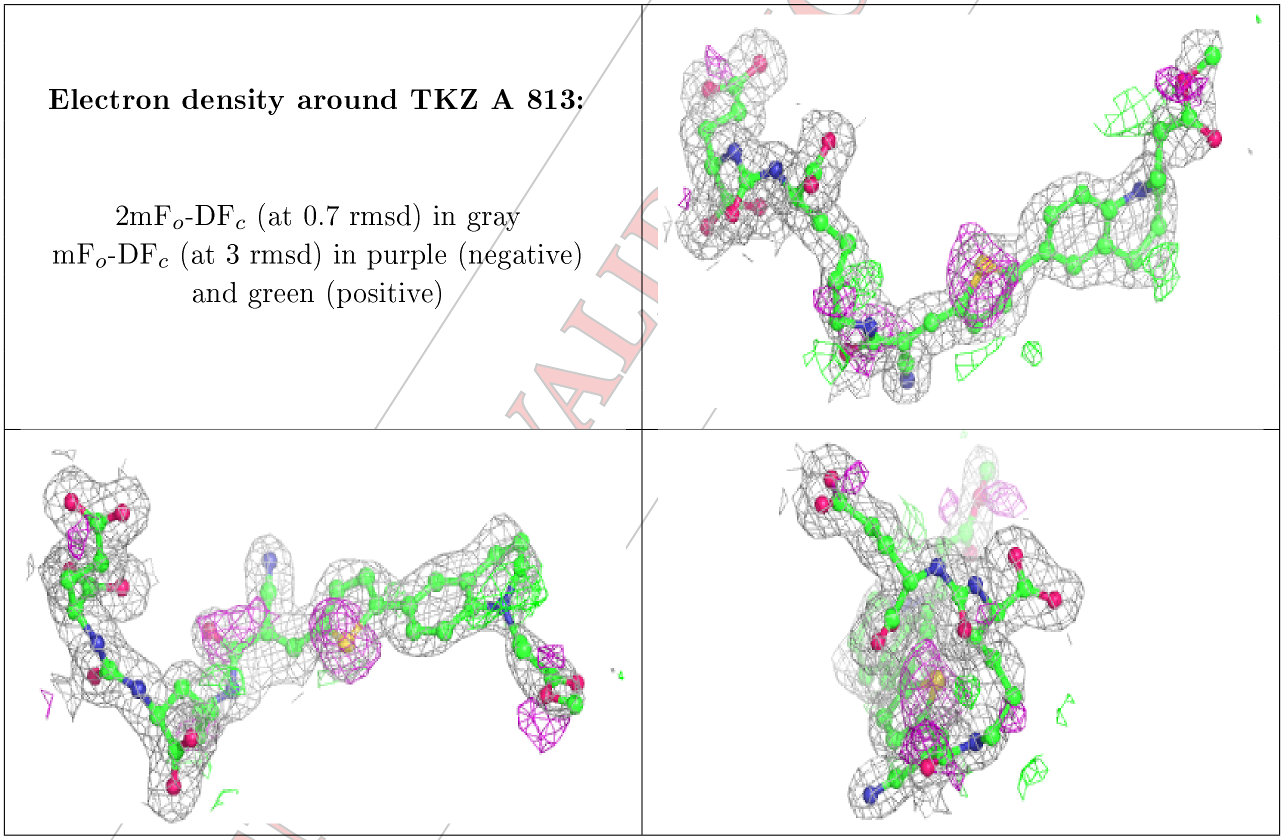

Electron density around TKZ A 813:

2mF$_o$-DF$_c$ (at 0.7 rmsd) in gray
mF$_o$-DF$_c$ (at 3 rmsd) in purple (negative)
and green (positive)

6.5 Other polymers (i)

There are no such residues in this entry.

CONFIDENTIAL VALIDATION REPORT
