# Supplementary material for: A prostate-specific membrane antigen activated molecular rotor for real-time fluorescence imaging
Source: Nat Commun. 2021 Sep 15;12:5460. doi: 10.1038/s41467-021-25746-6 (PMC8443597; doi:10.1038/s41467-021-25746-6)
Supplement: Supplementary file 6 — Description of Additional Supplementary Files [file 41467_2021_25746_MOESM6_ESM.pdf]

Description of Additional Supplementary Files

File Name: Supplementary Movie 1
Description: Real-time imaging of LNCaP treated with ODAP490. Images acquisition was performed as soon as the addition of fresh no-phenol 1640 medium containing 10 μM ODAP-490 to the cell culture dish, followed by real-time imaging for 2 hours at 1 frame per 3 minutes.

File Name: Supplementary Movie 2
Description: Real-time imaging of LNCaP treated with ODAP490 and CPZ. LNCaP cells were pretreated with 10 μM CPZ for 30 minutes. Images acquisition was performed as soon as the addition of fresh nophenol 1640 medium containing 10 μM ODAP-490 and 10 μM CPZ to the cell culture dish, followed by real-time imaging for 2 hours at 1 frame per 3 minutes.

File Name: Supplementary Movie 3
Description: Real-time imaging of LNCaP treated with ODAP490 and ZJ-43. LNCaP cells were pretreated with 100 μM ZJ-43 for 30 minutes. Images acquisition was performed as soon as the addition of fresh no-phenol 1640 medium containing 10 μM ODAP-490 and 100 μM ZJ-43 to the cell culture dish, followed by real-time imaging for 2 hours at 1 frame per 3 minutes.
